# Interferon-Based Biopharmaceuticals: Overview on the Production, Purification, and Formulation

**DOI:** 10.3390/vaccines9040328

**Published:** 2021-04-01

**Authors:** Leonor S. Castro, Guilherme S. Lobo, Patrícia Pereira, Mara G. Freire, Márcia C. Neves, Augusto Q. Pedro

**Affiliations:** 1 CICECO–Aveiro Institute of Materials, Chemistry Department, University of Aveiro, Campus Universitário de Santiago, 3810-193 Aveiro, Portugal; l.castro@ua.pt (L.S.C.); guilherme.lobo@ua.pt (G.S.L.); maragfreire@ua.pt (M.G.F.); 2 Centre for Mechanical Engineering, Materials and Processes, Department of Chemical Engineering, University of Coimbra, Rua Sílvio Lima-Polo II, 3030-790 Coimbra, Portugal; nunespereirapatricia@gmail.com

**Keywords:** Interferon, biopharmaceutical, recombinant DNA, production, purification, bioprocess development, formulation, excipient, drug delivery system, route of administration

## Abstract

The advent of biopharmaceuticals in modern medicine brought enormous benefits to the treatment of numerous human diseases and improved the well-being of many people worldwide. First introduced in the market in the early 1980s, the number of approved biopharmaceutical products has been steadily increasing, with therapeutic proteins, antibodies, and their derivatives accounting for most of the generated revenues. The success of pharmaceutical biotechnology is closely linked with remarkable developments in DNA recombinant technology, which has enabled the production of proteins with high specificity. Among promising biopharmaceuticals are interferons, first described by Isaacs and Lindenmann in 1957 and approved for clinical use in humans nearly thirty years later. Interferons are secreted autocrine and paracrine proteins, which by regulating several biochemical pathways have a spectrum of clinical effectiveness against viral infections, malignant diseases, and multiple sclerosis. Given their relevance and sustained market share, this review provides an overview on the evolution of interferon manufacture, comprising their production, purification, and formulation stages. Remarkable developments achieved in the last decades are herein discussed in three main sections: (i) an upstream stage, including genetically engineered genes, vectors, and hosts, and optimization of culture conditions (culture media, induction temperature, type and concentration of inducer, induction regimens, and scale); (ii) a downstream stage, focusing on single- and multiple-step chromatography, and emerging alternatives (e.g., aqueous two-phase systems); and (iii) formulation and delivery, providing an overview of improved bioactivities and extended half-lives and targeted delivery to the site of action. This review ends with an outlook and foreseeable prospects for underdeveloped aspects of biopharma research involving human interferons.

## 1. Clinical Importance of Interferon-Based Biopharmaceuticals and Market Overview

The lack of effective therapies for the treatment of a variety of human diseases has caused numerous health issues [1], representing the major driving force of Research and Development (R&D) activities toward the development of innovative medicines. In this regard, the emergence of biopharmaceuticals has allowed tremendous improvements in life quality [2], being at the cornerstone of the progress achieved in the last decades on the prevention and treatment of a wide range of diseases (e.g., cancer, infectious diseases, neurodegenerative diseases, among others). Biopharmaceuticals, also called biotherapeutics or biologicals, are products of biological origin such as proteins, nucleic acids, blood-derived products, somatic cells, or derivatives that are produced or extracted from living sources (e.g., microorganisms, cells, plants, or animals) [3,4,5]. Nowadays, recombinant therapeutic proteins and antibodies are considered the most abundant types of biopharmaceutical products in the market [3]. The success of biopharmaceutical-based therapies is linked to the development of recombinant DNA technology in late 1970s, which has allowed the large-scale production of human proteins and strongly stimulated systematic clinical investigations using new therapeutic approaches [6]. Following the approval of the first biopharmaceutical-insulin-in 1982, this market has been rapidly growing. According to the literature, from 2015 to 2018, approximately 112 biopharmaceuticals were approved in the United States of America (USA) and in the European Union (EU), essentially doubling the typical five-yearly historical approval pace and thus demonstrating the high demand for such products [3]. The overall growth of the biopharmaceutical market occurs due to two factors: the first one is related to the increment in the use of this type of product, and the second is closely related to the appearance of biosimilars [3,7,8]. Biosimilars are biological products similar to already existing medicines whose patents have expired [7,8], entering into the market with lower costs while exhibiting the same effects (quality, safety, and effectiveness) as the original biopharmaceutical [7]. Moreover, the global sales of therapeutic proteins have been increasing, being forecasted to increase on the approval of this type of therapeutic biomolecule in the coming years [3,7].

Among therapeutic proteins, the role of interferons (IFN) should be underlined, as they have been marketed for over 30 years with a considerable impact on the global therapeutic proteins market [3]. However, as recently highlighted by Timmerman [9] on the history of interferon’s trajectory, from the viral interference to the Hoffmann-La Roche product (Roferon A^®^, Hoffmann-La Roche, Basel, Switzerland), a series of obstacles had to be overcome-namely, restrictions to working with recombinant DNA, -to be in line with the interests of commercial partners and their demands for patent protection while addressing the desire by academic researchers working in the field for scientific outputs. IFN sales peaked between the 1980s and 2000s, as they were abundantly marketed and classified as “multiple drugs”, with an increasing range of therapeutic effects.

In a period of just six years, from 1986 to 1992, the world IFN market increased by approximately $740 million [10]. More recently, the global IFN market was valued at $6.9 billion in 2019, and it was estimated that it could grow to about $7.5 billion by 2020 due to an increasing demand for the use of IFNs along with antiretrovirals and antimalarial drugs in the treatment of SARS-CoV-2 disease (COVID-19) patients [11]. Furthermore, these projections are supported by the increasing incidence of chronic diseases, such as hepatitis B, hepatitis C, and multiple sclerosis, coupled with the use of IFNs in combinatorial therapies, the increasing adoption of IFN biosimilars with possible prophylactic or therapeutic effectiveness against virus pandemics, the advent of novel drug delivery systems, and continuous R&D activities involving IFNs [11]. Due to their relevance, several IFN products are currently in different stages of clinical trials. By January 2021, 172 active clinical trials involving the application of therapeutic IFN-based products were at different stages of development: 2 are in early phase 1, 50 in phase 1, 70 in phase 2, 28 in phase 3, and 6 in phase 4 of clinical trials [12].

The different clinical applications of IFNs and their corresponding marketed biological medicines are summarized in Table 1 [3,13,14]. Several IFN subtypes are well established in the market for the treatment of several pathologies, mainly in oncological treatment, as well as multiple sclerosis and chronic hepatitis C. To date, 21 formulations for the administration of IFN have received approval from EU and USA regulatory agencies, of which five have been withdrawn from the market—Infergen^®^ (Three Rivers Pharmaceuticals, Warrendale, PA, USA) in 2006 (EU), Roferon A^®^ (Hoffmann-La Roche, Basel, Switzerland) in 2007, Viraferon^®^ (Schering-Plough Corporation, Brussels, Belgium) in 2008 (EU), Albinterferon^®^/Albuferon^®^ in 2010 (Novartis, Basel, Switzerland; Human Genome Sciences, Rockville, MD, USA), and ViraferonPeg^®^ (Merck Sharp & Dohme Corp., Kenilworth, NJ, USA) in 2021 (EU). Rather than safety and efficacy issues, these products have been generally withdrawn from market due to requests of marketing authorization holders and the availability of similar products in market.

The commercialized formulations are produced mainly using *Escherichia coli* (*E. coli*) as host, except for Plegridy^®^ (Biogen Idec, Maidenhead, UK), Rebif^®^ (EMD Serono, London, UK), and Avonex^®^ (Biogen Idec, Maidenhead, UK), which are produced using Chinese hamster ovary (CHO) cells, and Alferon N^®^ (AIM ImmunoTech, Philadelphia, PA, USA) and Wellferon^®^ (Glaxo Wellcome, London, UK)), respectively, from human leukocytes and human lymphoblastoid cells. Moreover, some of the final products are available as PEGylated versions of IFNs, such as PegIntron^®^/Rebetol^®^ (Schering-Plough Corporation, Kenilworth, NJ, USA) combo pack, PEG-Intron^®^ (Merck Sharp & Dohme, Kenilworth, NJ, USA), ViraferonPeg^®^ (Merck Sharp & Dohme Corp., Kenilworth, NJ, USA), Intron A^®^ (Merck Sharp & Dohme Corp., Kenilworth, NJ, USA), and Plegridy^®^ (Biogen Idec, Maidenhead, UK), envisaging to enhance their stability and blood circulation half-life (addressed in Section 3.3.2).

Considering the relevance of IFNs for the treatment of several pathologies and their projected role in novel therapeutic regimens, as well as their essential role in improving patient health, this review article provides a comprehensive overview of the manufacturing of IFN-based biopharmaceuticals. The first section addresses the description of interferon characteristics, classification, and signaling pathways. The history and evolution of the manufacturing of IFNs are overviewed in the second section, subcategorized into the upstream stage, downstream stage, and formulation and delivery, in which representative works are outlined. An outlook is presented at the end of this work, complemented with foreseeable prospects for underdeveloped aspects of biopharmaceutical research and therapeutics involving IFNs.

## 2. Interferons Classification and Mechanisms of Action

In 1957, Isaacs and Lindenmann first saw a viral interference effect caused by bioactive material isolated from infected cells [15], thus assigning the term “*interferon*” to this interfering agent. Later, in 1978, due to improved molecular biology tools and developments on the upstream stage allowed researchers to obtain sufficient amounts of IFN with which to perform a reduced physical and chemical characterization of this biomolecule [16]. IFNs are natural cell-signaling glycoproteins produced by eukaryotic cells in response to viral infections, tumors, and other biological inducers, and thus represent part of the first line of defense of vertebrates against infectious agents [13,17].

IFNs cannot be classified as a single protein [16]; instead, they require use of different letters-α, β, and γ-to refer to the main classes of IFNs, which are, respectively, produced by leukocytes, fibroblasts, and lymphocytes (T cells and natural killer cells) [18]. In 1985, a new class (ω) was introduced in humans [19], and class τ [20] was further discovered in ovine cells. Furthermore, depending on their properties and their ability to bind to cell receptors, IFNs can also be classified into three different types (I to III), with each type displaying the ability to bind to a specific receptor and to trigger different signal transduction pathways and immunological responses, as shown in Table 2.

**Figure 1:**
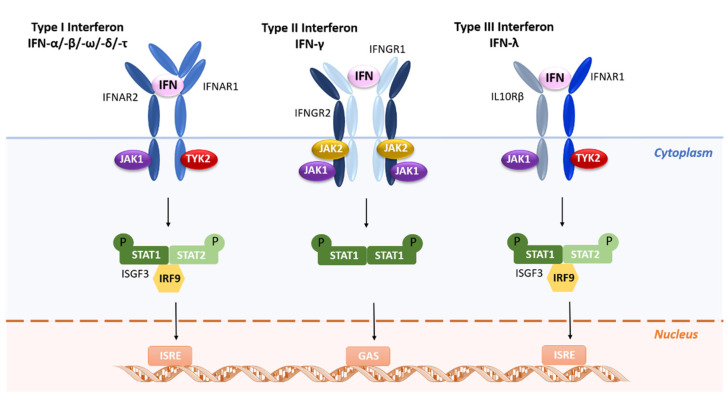
Receptor activation or ligand-receptor complex assembled by type I, type II, or type III interferons.

Briefly, type I IFNs bind to a heterodimeric receptor composed of two chains, IFNAR1 and IFNAR2, leading to the activation of the receptor-associated Janus-activated kinases (JAKs) TYK2 and JAK1, respectively (Figure 1) [22,23,24,25]. The next step in this signal transduction pathway is tyrosine phosphorylation of signal transducers and activators of transcription—STAT1 and STAT2—and the subsequent assembling of the heterotrimeric IFN-stimulated gene factor 3 (ISGF3) transcription factor complex. Distinctly, type II IFNs bind to a different cell-surface receptor consisting of IFNGR1 and IFNGR2 subunits, which in turn associate with JAK1 and JAK2, respectively, leading to phosphorylation of STAT1 (Figure 1) [26]. Finally, type III IFNs bind to a heterodimeric cytokine receptor composed of an IL-28R-binding chain and IL-10R2 that is shared with the IL-10 family of cytokines (Figure 1) [27]. The signaling cascade is like that of type I IFNs, in which the ISGF3 transcription factor complex binds to ISRE (IFN-stimulated response element) elements in gene promoters to induce transcription of IFN-inducible genes (ISGs). However, coordination and cooperation of multiple distinct signaling cascades, including the mitogen-activated protein kinase p38 cascade and the phosphatidylinositol-3-kinase cascade, are required for the generation of responses to IFNs [13].

Since their discovery by Isaacs and Lindenmann, IFNs have been known for their antiviral and antitumoral activities. These proteins own a broad spectrum of activity that impacts cellular metabolism and differentiation, and thus the antitumor effects appear to be due to a combination of direct antiproliferative effects, as well as indirect immune-mediated effects [16,17,28,29]. Accordingly, IFNs have been used in clinical practice to promote immune responses against infections and to treat autoimmune disorders and cancer, among others [16,17]. Furthermore, they can have synergistic or additive effects between them and with other biological response modifiers. The antiproliferative activity of IFN can be classified as direct or indirect [17,29,30], depending on if they inhibit the growth of cancer cells by stopping the cell cycle, apoptosis, or differentiation [17,30], or if they activate immune cells, such as T cells and natural killer (NK) cells, stimulating the immune system against oncogenesis and controlling tumor development [29,30]. The antiviral mechanism of IFN, like the antiproliferative mechanism, is based on the control of gene expression [17]. The antiviral response strongly depends on the virus, the host cell, and the type of IFN. The infection of a cell by a virus induces the production of IFN, which can then exert an autocrine or paracrine action on the surrounding cells. This phenomenon triggers the expression of proteins regulated by this IFN, which collectively constitute, in a very generalized way, the antiviral response responsible for inhibiting virus multiplication [17,28,31]. Schreiber and coworkers [32] determined the binding affinities (to isolated IFN receptor chains 1 and 2) and biological activity (antiproliferative and antiviral models) of IFNα subtypes. The authors found that the binding affinity and antiproliferative activity correlated with each other, but that for antiviral potency, there were several cases where the relationship appeared to be more complex than simple binding [32]. According to the authors, the concordance of the binding with the activity for most of the subtypes suggests that receptor binding events play a major role in the activity profiles of these molecules [32].

In sum, both the antiviral and antiproliferative mechanisms are based on the regulation of gene expression [28,30]. The proteins produced in response to the transcription and translation of these genes can have a direct or indirect action, leading in the latter case to the joint work of several aspects of the immune system [17,30]. Structural studies [33,34] have shown that type I IFNs consist of five α-helices (labeled A–E), which are linked by one overhand loop (AB loop) and three shorter segments (BC, CD, and DE loops) [23]. The detailed analysis of the structure of this subclass of IFNs revealed similar α-helical cores but large structural differences in AB loops. These insights demonstrate that subtle sequence differences and specific structural rearrangements influence the IFN-receptor interaction and may hold the key for the observed differences in biological activity [23]. Additional details on the structure of IFNs and their influence on IFN biological activities have been reviewed elsewhere [17,23,35,36,37].

## 3. Therapeutic Cloned Interferons

Commercial IFN-based products were first derived from leukocytes and then from lymphoblastoid lines [36]. However, as both protein extraction from natural producers and chemical synthesis undergoes inherent constraints that limit regular large-scale production, recombinant DNA technologies have rapidly become a choice for therapeutic protein production, including IFNs [38]. The relatively small size (Mw ~20 kDa) and compactness of the IFN protein, combined with the lack of any functional glycosylation (at least in some cases, unglycosylated IFNs are predicted to be functionally identical to their glycosylated counterparts), has contributed to high yield and improved bioactivity [36]. These therapeutic proteins are obtained ex vivo mostly in biological systems and must guarantee, in addition to full protein functionalities, a cost-effective industrial manufacturing in the absence of impurities (host cell proteins, DNA, aggregates, among others) [38].

**Figure 2:**
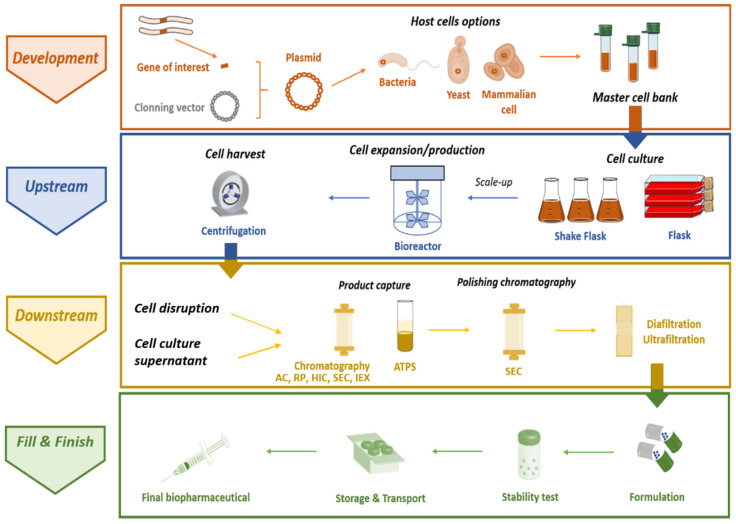
Overview of the manufacturing of IFN-based biopharmaceuticals (ATPS–Aqueous two-phase system; AC–Affinity chromatography; IEX–Ion-exchange chromatography; HIC–Hydrophobic interaction chromatography; RP–Reverse phase chromatography; SEC–Size exclusion chromatography).

The complete manufacturing process to obtain recombinant therapeutic proteins comprises four main stages, summarized in Figure 2: (i) the development stage, in which the gene of interest is isolated, cloned in a suitable plasmid, and then the recombinant plasmid is introduced in the selected host, allowing the master cell bank to be obtained; (ii) the production itself, or *upstream* stage, which is associated with the choice of a particular expression system and respective culture conditions; (iii) the *downstream* stage, including the recovery of the target protein, followed by its purification from a heterogeneous and highly complex matrix that generally encompasses chromatographic techniques (corresponding to the most expensive part of the process); and (iv) fill and finish, whereby the final product formulation is developed according to the method of administration, and the process must ensure that the stability and biological activity of the purified biopharmaceutical is maintained during storage and transport [4,39]. Protein drugs must necessarily conform with quality constraints stricter than those expected in the production of enzymes for chemical industries, which consequently defines the choice of recombinant hosts, protocols, and production/purification strategies [38]. Moreover, there is a generic consensus about the need to enable drugs for cell- or tissue-targeted delivery, aiming for a reduction in dosage, production costs, and side effects [38]. To this end, therapeutic proteins are usually administered in formulations whose compositions are optimized to guarantee improved stability and delivery of target biopharmaceuticals. In general, the purity, activity, and safety of the finished products are ensured by critical aspects, including host cell development, cell culture, cell bank establishment, protein synthesis, purification process, and subsequent protein analysis, formulation, storage, and handling [40].

### 3.1. Upstream Stage

Recombinant biopharmaceuticals production requires the optimization of the *upstream* stage either by adapting the host, the vector, or the promotor employed or by changing the conditions applied for host growth. Usually, the choice of a host is the first step to consider and depends on the type of protein being produced—namely, which eukaryotic systems are more suitable with regard to the need for extensive post-translational modifications. Moreover, the economic viability of the bioprocess is also highly dependent on the selected host [41,42]. *E. coli* and *Pichia pastoris* (*P. pastoris*, currently reclassified as *Komagataella pastoris*) microfactories are the most widely used hosts to obtain IFNs for clinical applications and with which to perform structural and functional studies. With the exception of IFNβ-which in addition to *E. coli* is also produced from CHO cell lines and IFNα-n3 (Alferon N^®^) and IFNα-n1 (Wellferon^®^), respectively, obtained from human leukocytes and lymphoblastoid cells-all other commercialized IFN formulations are based on *E. coli* [3]. Since the *in vivo* efficacy of IFNβ increases by its natural glycosylation [43], mammalian cell lines are the best host with which to obtain recombinant proteins with native glycosylation patterns, and thus represent the best compromise between yield and quality. Although since the early 1980s, several hosts have been applied to produce all classes of IFN molecules (e.g., in addition to the above-mentioned, insect cells, plants, transgenic mice, among others), in this review the application of *E. coli* and *P. pastoris* to produce recombinant IFN molecules is detailed given their improved performance and widespread use.

#### 3.1.1. Expression Using *Escherichia coli*

*E. coli*-based systems are often the preferred choice for recombinant proteins production, mostly due to the well-known advantages, such as the unparalleled fast growth kinetics (growth can double in about 20 min in appropriate conditions), easy manipulation, simple culture requirements, and cost-effectiveness [39,42,44]. Even though the recombinant protein production process can be a metabolic burden for the microorganism, causing a decrease in the generation time, high cell-density cultures can also be obtained using the host *E. coli*. Moreover, the media necessary for their growth is made of inexpensive and readily available components, such as glucose, peptone, yeast extract, or sometimes even the commercially available Luria–Bertani (LB) medium [45,46,47]. From gene cloning to protein purification, the cellular and molecular tools required for protein expression based on *E. coli* are readily available. However, failures to obtain a functional recombinant protein are not uncommon, which have stimulated continuous research in this regard. Major improvements achieved from 2014 to 2019 were recently reviewed by Ceccarelli and coworkers [44].

The expression of proteins outside their original context can pose additional constraints since they might contain codons that are rarely used in the desired host or contain expression-limiting regulatory elements within their coding sequence [42]. In the case of IFN, this is particularly significant for its expression in *E. coli* since the presence of clusters AGG=AGA, CUA, AUA, GGA, or CCC codons in heterologous genes can decrease the quantity and quality of the heterologous protein [48]. Indeed, distinct studies [46,49,50,51] have shown that removing codon bias while using codon-optimized versions of target genes (codon usage such as *E. coli*) can successfully increase the production of soluble IFN molecules in *E. coli*.

In general, IFN expression in *E. coli* has been accomplished using different strains, with special emphasis on BL21 strains [45,46,47,52,53,54], first described by Studier in 1986 after various modifications of the B line, and like other parental B strains, these cells are deficient in the Lon protease, which degrades foreign proteins [55]. In some cases, strains such as Origami B [56] and SHuffle [57], with improved ability to increase disulfide bond formation of proteins in cytoplasm, are also applied.

The *lac* promoter is widely studied and important in prokaryotic systems. It is a crucial component of the *lac operon*, being induced by lactose that can also be used for protein production [39]. However, induction is difficult in the presence of readily metabolizable carbon sources such as glucose [39]. On the other hand, the T7 promoter system present in pET vectors is also extremely popular for recombinant protein expression. In this system, the gene of interest is cloned behind a promoter recognized by the phage T7 RNA polymerase (T7 RNAP). This highly active polymerase should be provided in another plasmid or, most commonly, it is placed in the bacterial genome (as is the case of BL21 (DE3) strains) in a prophage (λDE3) encoding for the T7 RNAP under the transcriptional control of a lacUV5 promoter. Thus, the system can be induced by lactose or its analog, isopropyl *β-D*-1-thiogalactopyranoside (IPTG) [39]. Using *E. coli* BL21-SI, whereby T7 polymerase is under the control of a salt-inducible promoter, strategies using NaCl as inducer have been reported [46,54,58].

The high success displayed by *E. coli* for the expression of recombinant IFN molecules is associated with IFN’s relatively small size and lack of any functional glycosylation for some IFNs [36]. However, it should be remarked that the *in vivo* efficacy of IFNβ increases by its natural glycosylation profile [43]. As summarized in Table 3, this sub-section comprises representative examples of the expression of distinct classes of IFN molecules in *E. coli*, including (i) expression in periplasm and in the form of inclusion bodies; (ii) engineered strategies to increase IFN solubility, allowing their soluble expression in cytoplasm; (iii) optimized culture conditions toward enhanced production and stability; and (iv) strain engineering.

**Table 3:** Representative studies of the expression of therapeutic IFNs in *Escherichia coli* recombinant systems.

IFN Type	Strain/Vector	Promotor	Culture Media	Antibiotic	Inducer	Production Scale	Expression	Level of Expression
IFNα-2, IFNα-8 [59]	BL21(DE3)-RIL pGEM-T	T7 *lac*	LB + 1% glucose	Ampicillin	IPTG (1 mM)	Shake-flask	Intracellular (IB)	70.0 mg/L IFNα-2 and 75 mg/L IFNα-8 (refolded IB)
Hybrid IFNs [59]	BL21(DE3)-RIL pET-16b							70.0 mg/L IFNα-828 (refolded IB)
IFNα [46]	BL21-SI pAE	*proU*	LB without NaCl	Ampicillin	NaCl (0.3 M)	Shake-flask	Intracellular (Soluble)	75.0 mg/L (native) 210 mg/L (6xHis-tagged)
IFNα-2b (GST-fusion) [56]	Origami B pGEX4T1	*tac*	LB	Ampicillin	IPTG (0.1, 0.5, 1 mM)	Shake-flask	Intracellular (Soluble)	100 mg/L (purified)
IFNα-2b [60]	JM109(DE3) pET-9	T7	Glucose; yeast extract; K_2_HPO_4_; KH_2_PO_4_; (NH_4_)_2_SO_4_; MgSO_4_	Kanamycin	IPTG (1 mM)	Shake-flask; 5L Fermenter	Intracellular (IB)	13.8 mg IFNα-2b per gram wet cells
IFNβ [58]	BL21-SI pTPM13	T7	Glucose; K_2_HPO_4_; KH_2_PO_4_; (NH_4_)_2_SO_4_; MgSO_4_; thiamine	Ampicillin	NaCl (0.3 M)	Shake-flask	Intracellular (IB)	61.0 mg/L
IFNβ-1b [47]	BL21 (D3)	T7	TB	Ampicillin	IPTG (0.2 mM)	Bioreactor (2L)	Periplasmatic	255 mg/L
IFNε [45]	DH5α pBV220	T7	LB	Ampicillin	42^o^C	Shake-Flask	Intracellular (IB)	8.00 mg/L (purified)
IFNγ [54]	BL21-SI (pBAL0; pBAL1; pBAL3)	N/A	Glucose; KH_2_PO_4_; (NH_4_)_2_SO_4_; MgSO_4_; thiamine	Ampicillin	NaCl (0.3 M)	Shake-Flask	Periplasmatic	45.0 mg/L (post-induction temperature = 20.0 °C)
IFNγ [53]	BL21 (DE3) pET14b	T7	LB M9YE TB	Ampicillin	IPTG (1 mM)	Shake-flask	Intracellular (IB)	140 mg/g DCW (TB) 130 mg/g DCW (LB) 115 mg/g DCW (M9YE)
						Bioreactor (1L)		182 mg/g DCW (TB) 170 mg/g DCW (LB) 160 mg/g DCW (M9YE)
IFNγ [52]	BL21 (DE3) pET3a	*lac*	M9 modified medium contained (glucose, K_2_HPO_4_, KH_2_PO_4_, C_6_H_8_O_7_, (NH_4_)_2_SO_4_, MgSO_4_)	NR	IPTG (2.25 mg/g/L per DCW)	Bioreactor (1L)	NR	51.0 × 10^3^ mg/L
IFN-con [57]	SHuffle Champion™ pET SUMO	T7*lac*	TB	Kanamycin	IPTG (0.1, 1 mM)	Shake-Flask	Intracellular (Soluble)	50.0 mg/L (Purified)

Abbreviations: DCW–Dry cell weight; IB–Inclusion bodies; IPTG-Isopropyl B-D-1-thiogalactopyranoside; TB—Terrific Broth; LB—Luria–Bertani medium; NR–Not Reported.

Although the production yield associated with the periplasmic pathway is lower [61], this route presents several advantages, as follows: (i) lower proteolysis; (ii) low amount of contaminating proteins; (iii) correct formation of disulfide bonds; (iv) correct folding; and (v) simple methods for releasing the target protein [62]. Secretion of IFN molecules to *E. coli* periplasm was achieved by fusing a signal peptide to the *N*-terminal residue and was investigated by the Rodríguez [54] and Vahidi [47] research groups. IFNγ was expressed using *E. coli* BL21-SI and three different expression vectors-namely, pBAL0, pBAL1, and pBAL3 [54]. Envisaging to transport IFN to the periplasm of host cells, the synthetic IFNγ gene was fused to SP1 and SP3-two Sec-dependent artificial signal peptides: SP1 signal peptide was fused to synthetic IFNγ gene to obtain the expression vector pBAL1; and the SP3-IFNγ gene was obtained by polymerase chain reaction (PCR) using plasmid pBAL1 as a template and then subcloned in pET12a to generate the expression vector pBAL3. A construction without signal peptide, named pBAL0, was constructed by PCR using the plasmid pBAL1 as a template. Protein expression was induced using 0.3 M NaCl. Initial experiments showed that SP1-IFNγ and SP3-IFNγ were processed completely (no precursor detected) when cells were cultivated using minimal medium and a post-induction temperature of 32.5 °C. The SP3 signal peptide was more efficient than SP1 for the secretion of IFNγ, and approximately 60.0% of total IFNγ was secreted to the periplasm using SP3 and a post-induction temperature of 20 °C [54]. Vahidi and collaborators [47] studied the optimal fermentation conditions for periplasmic expression of IFNβ-1b in shake-flasks whilst keeping the acetate excretion at the lowest amount; subsequently, the conditions yielding the best results were exploited for IFNβ expression in a benchtop bioreactor. *E. coli* BL21 F- *ompT hsdS*_B_ (rB-mB-) *gal dcm* (D3) transformed with a plasmid that contained the strong inducible T7 promoter under the control of *lac*-operator sequence, which was used as the host to produce IFNβ-1b. The *N*-terminal pelB signal sequence was fused to the IFN gene for periplasmic localization. The transformed bacteria were inoculated in Terrific Broth (TB) medium, and the maximum expression occurred with the following fermentation conditions: 7.81 g/L glucose, optical density (OD) at 600 nm prior induction of 1.66, and induction temperature of 30 °C, achieving yields of 0.255 g/L in a 2 L bioreactor [47]. These two works show that IFN secretion to periplasm can be achieved by fusing the target gene to distinct signal sequences and that the yields of secreted IFN strongly depend upon culture conditions—namely, temperature and culture medium.

In addition to the secretion to the periplasm or as soluble cytosolic proteins, proteins can be intracellularly produced in *E. coli* in the form of inclusion bodies, usually as biologically inactive proteins. Although requiring additional solubilization and refolding steps (addressed in detail in Section 3.2.1), these insoluble cytoplasmic aggregates can be produced in high concentration, so that the amount of generated product often outweighs the additional downstream steps and can boost time/space yields for recombinant protein production [63]. Additional advantages of inclusion body formation include, among others: (i) easy separation from bacterial cytoplasmic proteins by centrifugation; (ii) the production of proteins toxic to the cell; and (iii) protection of the heterologous protein against proteolytic enzymes [60,61,62]. Platis and Foster [59] reported the expression of IFNα-2, IFNα-8, and their hybrids as inclusion bodies into BL21(DE3)-RIL *E. coli* cells, which were modified as follows: the pGEM-T vector regulated by T7 *lac* promoter was applied for IFNα-2 and IFNα-8 (pGEM-IFNα-2 and pGEM-IFNα-8) expression, while pET-16b was used for the production of hybrid IFNs. Aiming to optimize the yield of IFN using IPTG as inducer, BL21-RIL(DE3) cells were cultivated at different temperatures (25, 30, and 37 °C) in LB medium, and it was observed that by reducing the temperature to 25 °C, a maximum yield of IFN was obtained after 6 h (Table 3) [59]. Another study by the Prazeres research group [60] explored the production of IFNα-2b using the strain *E. coli* JM109 (DE3) transformed with the vector pET-9a (pET9-IFN-MR1). Batch fermentation was performed in a 5 L fermenter at 37 °C, pH 7.0, and the dissolved oxygen was kept at a set point of 30% air saturation. Cells were grown in a complex medium and recovered at 20 h post-induction with IPTG (stationary phase), from which an induced level of 13.8 mg total IFNα-2b per gram wet cells were obtained [60]. Overall, these two studies represent successful examples of IFN expression as inclusion bodies in *E. coli* using shake-flasks and fermenters, which were obtained with biological activity after suitable recovery protocols.

Envisaging to avoid the formation of inclusion bodies, some approaches that allow the expression of soluble IFNs in the *E. coli* cytoplasm have been reported. Fathallah and collaborators [56] employed a dual strategy for improving the expression of soluble IFNα-2 in *E. coli*. On one hand, a recombinant expression plasmid (pGEX-D-IFNα-2b) was constructed, in which the IFNα-2b cDNA was fused with the glutathione S-transferase (GST) coding sequence downstream of the *tac*-inducible promoter. On the other hand, the expression of soluble IFNα-2 as GST fusion protein was performed in Origami B (*trxB^−^/gor^−^)* and BL21 *(lon^−^/ompT^−^) E. coli* strains in LB medium, under optimized environmental factors such as culture growth temperature and inducer (IPTG) concentration [56]. The choice of *E. coli* Origami B as an alternative host was dictated by the fact that proper folding of the IFN molecule requires the formation of two disulfide bridges between Cys_1_–Cys_98_ and Cys_29_–Cys_138_. Indeed, in the cytoplasm of normal *E. coli* strains, cysteines are actively kept in the reduced state by a pathway involving thioredoxin reductase and glutaredoxin [64]. Disruption of the *trxB* and *gor* genes, encoding the two major reductases of *E. coli*, allows the formation of disulfide bonds in the *E. coli* cytoplasm [65]. The amount of soluble IFNα-2b using *E. coli* BL21 strain was superior at 25 °C using 0.1 or 0.5 mM IPTG for induction, as compared with growth at 37 °C and 1 mM IPTG [56]. The expression of the soluble GST-IFNα-2b protein was increased more than 2-fold (a yield of 100 mg/L) when expressed at 25 °C and 0.5 mM IPTG using Origami B host strain. This study demonstrates that high yield production of soluble and functional IFNα-2b tagged with GST can be achieved in *E. coli* [56]. Another strategy by Laurine and collaborators [57] explored the production of soluble IFNα-2 consensus using the SHuffle™ *E. coli* strain that promotes the expression of proteins with disulfide bonds. An IFN-consensus gene was cloned into the Champion™ (Thermo Fisher Scientific^®^, Waltham, MA, USA) pET SUMO expression vector downstream of the SUMO fusion partner (SUMOIFN-con fusion protein), herein acting as a solubility enhancer, and a yield of 50.0 mg/L was obtained [57]. In 2016, Chloe and coworkers [51] performed a comparative study to evaluate the expression levels and solubilities of IFNα-2b in *E. coli* BL21 (DE3) strain using a wide range of fusion partners. Seven fusion tags—thioredoxin (Trx), hexahistidine (6x His), maltose-binding protein (MBP), N-utilization substance protein A (NusA), protein disulfide isomerase (PDI), GST, and b’a’ domain of PDI (PDIb’a’)—were evaluated for soluble overexpression of codon-optimized IFNα-2b at two different expression temperatures, 37 and 18 °C [51]. Apart from GST fusion, the expression levels of all tagged IFNα-2b constructs increased at lower temperatures (18 °C). At 37 °C, all the constructs demonstrated poor solubility, and most of the protein was found in the insoluble cell pellet fraction. However, IFNα-2b solubility was markedly improved for Trx, PDIb’a’, MBP, PDI, and NusA-based constructs at the lowest temperature in study. Considering the MBP construct’s expression level, solubility, and small tag size, this fusion partner was selected to be further applied for chromatography-based purification processes [51]. Nascimento and coworkers [46] produced two genes of IFNα: one containing the native DNA sequence and the other with a mutated form in which two cysteine amino acid residues were replaced by serines (at positions 1 and 98) in an attempt to improve the stability of the protein. In this case, DNA sequences were cloned into pAE, an *E. coli* vector that allows heterologous protein expression with or without a histidine tag using the *E. coli* BL21 (SI) strain. The media employed was 2YTON-amp (LB medium without NaCl), and the bacteria were grown overnight at 30 °C. The production of recombinant proteins was achieved by the addition of NaCl to the medium, and the resulting yield was 75.0 and 210 mg/L for the proteins without and with a 6xHis-tag, respectively. Moreover, the authors claimed that the mutated form of His-tagged IFN exhibited a slightly higher antiviral activity when compared to their native His-tagged counterpart, further suggesting that the mutation can increase the stability of IFN [46]. In general, these studies show the beneficial effect of using improved strains and solubility enhancers as fusion partners to increase the expression of soluble IFN molecules in *E. coli*, although it should be remarked that, if required, suitable protocols for tag removal must be implemented [56].

The yield of recombinant proteins can be highly enhanced through the optimization of culture conditions, such as medium composition, inducer concentrations, cell density at the moment of induction, and post-induction period, among others. In 2007, Rodríguez and coworkers [58] optimized the production of IFNβ using the strain *E. coli* BL21-SI and the pTPM13 vector with the T7 promoter. Aiming for the highest IFNβ production, the authors used response surface methodology and a Box–Behnken design to optimize several parameters-namely, culture medium, temperature, cell density, and inducer concentration. This study [58] was the first report to demonstrate the successful performance of the BL21-SI system in a minimal medium (containing glucose, ammonium hydrogenphosphate, potassium dihydrogenphosphate, magnesium sulfate, and thiamine) for IFNβ production. The maximum level of IFNβ production—61.0 mg/L—was attained with the following conditions: temperature of 32.5 °C, cell density of 0.64, and inducer concentration of 0.3 M NaCl [58]. A distinct study by Maghsoudi and collaborators [52] evaluated the expression of IFNγ using *E. coli* BL21 (DE3) in a host modified with the pET3a vector under several operational parameters-namely, the amount of IPTG (ranging from 0.565 to 22 mg/g/L at seven levels), cell density at induction time (53, 65 and 75 g (dry cell weight, DCW)/L), and the length of the interval of post-induction (3, 4, and 5 h after induction time) for the production. Fed-batch cultivation was performed with M9 modified medium, and the following optimum conditions were identified: 2.25 mg/g/L IPTG per DCW, DCW = 65 g/L at induction time, and a post-induction interval of 4 h [52]. Using these conditions, the final concentrations of biomass and IFNγ reached, respectively, 127 g/L (DCW) and 51x10^3^ mg/L of IFNγ after 17 h, and the final specific yield and overall productivity obtained were 0.4 g IFNγ/g DCW and 3 g IFNγ/L/h, respectively. The increase in the level of overall productivity could be due to: (i) recombinant protein production under induction optimum conditions; (ii) reduction of process time; (iii) increase in plasmid stability; (iv) decrease in accumulation of by-products, especially acetate; (v) presence of nutrients (glucose, ammonium and phosphate) at a suitable concentration range during fed-batch cultivation; and (vi) higher ribosome content at higher growth rates [52].

Mukherjee and collaborators [53] performed different continuous cultures to understand the IFNγ formation kinetics in *E. coli* BL21 (DE3) modified with the T7 promotor-based pET14b vector at different dilution rates and media. Growth was performed with constant agitation at 200 rpm at 37 °C using three different media: LB (10 g/L bacto tryptone, 5 g/L yeast extract, 5 g/L NaCl); M9YE (0.5 g/L NaCl, 1 g/L NH_4_Cl, 3 g/L K_2_HPO_4_, 0.1 mL/L 1 M CaCl_2_, 2 mL/L 1 M MgSO_4_, 0.2% glucose, and 0.2% yeast extract), and TB (24 g/L yeast extract, 12 g/L tryptone, 0.4% glycerol, 2.31 g/L KH_2_PO_4_ and 12.54 g/L K_2_HPO_4_). At the shake-flask level, the amount of IFNγ produced was quantified by ELISA, and the maximum Y_p/X_ was found to be 140, 130, and 115 mg/g DCW for TB, LB, and M9YE, respectively [53]. As TB is a highly enriched medium, the final OD_600_ reached 8.5 and thus may explain the obtained results; hence, the volumetric product concentration also exceeded the other two media by >3.3-fold. In turn, the maximum Y_p/X_ value reached in the continuous culture studies in a 1 L bioreactor after 6 h post-induction was, respectively, 182, 170, and 160 mg/g DCW for TB, LB, and M9YE [53]. Ebrahimi and coworkers [66] investigated the susceptibility of IFNγ against oxidative stress during fermentation in *E. coli*, in which the carbonyl content was taken to be an indicator of protein oxidation. To this end, cultivations were performed at 5, 30, and 60% dissolved oxygen; the carbonyl content showed no significant increase at 5 and 30% dissolved oxygen, but a 10-fold increase was observed at 60% dissolved oxygen. This study points out that lowering oxygen tension can minimize oxidized forms of IFNγ and avoid the formation of product-related impurities that are very similar to the target product and thus contribute to increased IFNγ biological activity [66]. As with other proteins, these studies demonstrate that refining the culture conditions contributes to increasing the yield and quality of IFNs expressed in *E. coli*.

**Figure 3:**
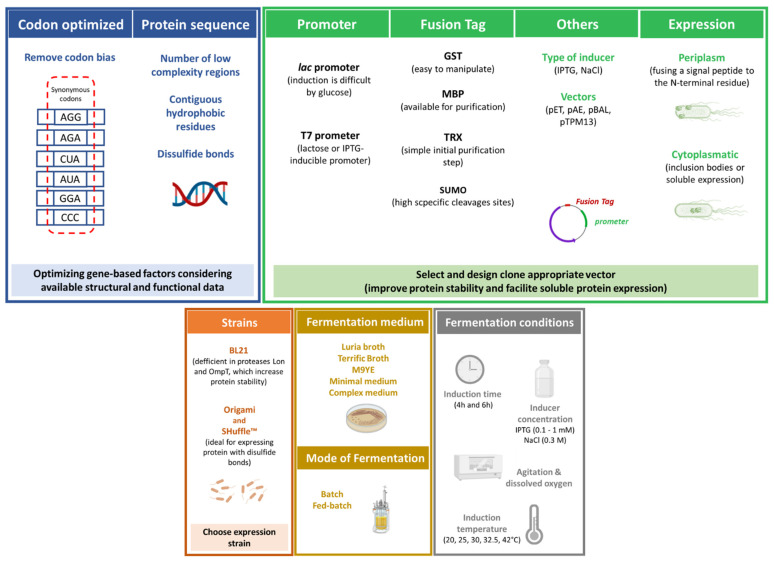
Optimized factors towards enhanced expression of IFNs using recombinant *E. coli.*.

Originally developed from wildtype K12 strain MG1655 for increased plasmid yield, the *E. coli* phosphoglucose isomerase (pgi) mutant strain GALG20 was recently applied in IFNγ production by Prazeres and collaborators [67]. The authors found that pgi deletion increases amino acid biosynthesis and flux efficiency toward IFNγ synthesis by 11%. To confirm the in silico metabolic network predictions, the authors determined the specific IFNγ yields and found that GALG20 (DE3) produced 3-fold and 1.5-fold more IFNγ as compared with MG1655(DE3) and BL21(DE3), mostly obtained in the form of inclusion bodies for all strains [67]. As with several commercially available strains, this study reinforces that continuous improvements in *E. coli* strains contribute to increasing the yield and quality of recombinant IFN molecules. Figure 3 summarizes optimized factors leading to enhanced expression of IFNs using recombinant *E. coli*.

#### 3.1.2. Expression Using *Pichia pastoris*

The success of *P. pastoris* as a host for recombinant IFN production is due to its ability to achieve high cell densities, giving higher expression levels of heterologous proteins with some post-translational modifications not achievable with *E. coli*-based systems [68]. Moreover, in comparison with other eukaryotic systems, *P. pastoris* is regarded as being faster, easier to use, and less expensive than expression systems derived from higher eukaryotes, such as insect and mammalian tissue culture cell systems [68,69]. As a methylotrophic yeast, *P. pastoris* is capable of metabolizing methanol, with alcohol oxidase (AOX) being the strong and most widely used promoter for recombinant protein expression using methanol as an inducer [68]. Depending on the deletion of one or two genes encoding AOX, *aox1* and *aox2*, *P. pastoris* can present different phenotypes, which should be considered when selecting the culture conditions, particularly regarding the concentration of methanol [70]. In addition to AOX, other inducible or constitutive promoters can be applied, such as the GAP (glyceraldehyde-3-phosphate dehydrogenase) promoter that relies on the constitutive expression of glyceraldehyde-3-phosphate dehydrogenase enzyme by *P. pastoris* [68].

With *P. pastoris*, heterologous proteins can either be expressed intracellularly or secreted into the culture medium as long as a sequence signal is introduced upstream of the target gene; since only low levels of endogenous proteins are secreted, the subsequent purification steps are generally more straightforward [71]. However, secreted proteins such as IFNs may be unstable in the culture medium, being readily degraded by proteases, a problem that can be overcome e.g., using protease-deficient strains (SMD 1168) [68] or by supplementing the culture medium with casamino acids [72]. A series of commercially available (ThermoFisher Scientific^®^, Waltham, MA, USA)) plasmids is commonly used for intracellular (pPICZ or pGAP) and secreted (pPICZα or pGAPα) recombinant protein expression under the control of AOX promoter, in which the latter encompasses the *Saccharomyces cerevisiae* (*S. cerevisiae) α*-factor prepro signal sequence [71]. Other plasmids for secretory (pPIC9K, pHIL-S1, pGAPZα, pJL-SX, pBLHIS-SX) and intracellular expression (pPIC3.5K, pHIL-D2, pGAPZ, pJL-IX) are based on different promoters and gene markers [73]. Due to the high ability displayed by *P. pastoris* to secrete heterologous proteins, usually relying on AOX promoter, this sub-section encompasses representative examples of *P. pastoris* bioprocesses involving IFN secretion driven by AOX, as summarized in Table 4.

**Table 4:** Representative studies of the expression of therapeutic IFNs in *Pichia pastoris* recombinant systems.

IFN Type	Strain/Plasmid	Promoter	Media	Antibiotic	Inducer	Scale	Type of Expression	Level of Expression
IFNα-2b [74]	GS115 pPICZα	AOX	BMGY/BMMY	Zeocin	Methanol	Shake-flask	Secreted (αprepro)	450 mg/L
IFNα-2b [72]	KM71H pPICZα-hIFNα-2b	AOX1	BMGY/BMMY	Zeocin	Methanol	Bioreactor	Secreted (αprepro)	600 mg/L
IFNα-2b [75]	GS115 pPIC9HSS pPIC9IFN pPIC9αIFN	AOX	BMG/BMM	Ampicilin	Methanol	Shake-flask	Secreted (mutated αprepro)	200 mg/L (pPIC9αIFN)
IFNα-2b [76]	GS115 pPIC9KN	AOX	BMGY/BMMY	Geneticin	Methanol	Bioreactor	Secreted (αprepro)	300 mg/L
IFNγ [77]	GS115 pPICZαA	AOX	BMGY/BMMH	Zeocin Geneticin	Methanol	Shake-flask	Secreted (αprepro)	2.50 mg/L
IFNα-2b [78]	Glycoswitch^®^ *P. pastoris* SuperMan5	AOX1	BMGY/BMMY	NR	Methanol	Shake flask	Secreted (N/A)	436 mg/L
N-glycosylated IFNβ-1 [79]	GS115 pPIC9IFN	AOX1	BMGY/BMMY	NR	Methanol	Shake-flask	Secreted (αprepro)	6.00–12.0 mg/L
IFNλ [69]	GS115 pAO815	AOX	BMG/BMM	Ampicilin	Methanol	Shake-flask	Secreted (αprepro)	65.0 mg/L
rHSA/IFNα-2b [80]	N/A pPIC9	AOX	N/A	NR	Methanol	Biostat C 15L fermenter	Secreted (HSA signal peptide)	250 mg/L

Abbreviations: AOX–Alcohol oxidase; NR–Not reported.

In 2007, Cheng and coworkers [74] employed the *P. pastoris* GS115 strain modified with the pPICZα vector to produce IFNα2b. The transformants were grown on buffered glycerol-complex medium (BMGY, composed of 1% (*w/v*) yeast extract, 2% (*w/v*) peptone, 1.34% yeast nitrogenous base (YNB), 1% glycerol, 0.4 mg/mL biotin in 0.1 M potassium phosphate buffer, pH 6.0). To induce expression, the cell pellet was then resuspended in buffered methanol-complex medium (BMMY, BMGY with 0.5% methanol instead of 1% glycerol) in a 1 L flask and grown at 20 °C with shaking. SDS-PAGE and Western-blotting assays of culture broth from a methanol-induced expression strain demonstrated that recombinant IFNα-2b was secreted into the culture medium. The expression level of IFNα-2b was estimated to be about 450 mg/L culture in a fed-batch mode. Moreover, the authors found that decreasing the temperature from 30 to 20 °C during the methanol feed phase increased the yield of the recombinant protein, as the levels of extracellular proteases were reduced [74]. Kallel and collaborators [72] optimized the volumetric productivity of IFNα-2b using fed-batch cultivations of *P. pastoris* KM71H in a 5 L bioreactor. To this end, the composition of the media used for bioreactor cultures were as follows: the batch medium contained 40 g/L glycerol, 18.2 g/L K_2_SO_4_, 7.28 g/L MgSO_4_, 4.13 g/L KOH, 0.93 g/L CaSO_4_·2H_2_O, 85% orthophosphoric acid (26.7 mL/L), 5 mL/L basal salts of fermentation PTM1, and 0.2 g/L biotin (2 mL/L); the fed-batch medium contained the same components, with the exception of glycerol (450 g/L), supplemented with PTM1 (8 mL/L) and 0.2 g/L biotin (5 mL/L). The PTM1 solution contained: 6 g/L CuSO_4_·5H_2_O, 0.08 g/L NaI, 3 g/L MnSO_4_·H_2_O, 0.2 g/L Na_2_MoO_4_·2H_2_O, 0.02 g/L H_3_BO_3_, 0.5 g/L CoCl_2_, 20 g/L ZnCl_2_, 65 g/L FeSO_4_·7H_2_O, 0.2 g/L biotin, and 5 mL/L H_2_SO_4_ 98%. The methanol fed-batch solution contained 987 mL/L methanol, 500X biotin (5 mL/L) and 8 mL/L PTM1. The authors found that IFNα-2b was highly sensitive to proteases activity during high cell density culture, i.e., IFNα2b was totally degraded 20 h after starting methanol feeding. To improve IFNα-2b expression and prevent its proteolysis, the replacement of culture medium with fresh medium after glycerol fed-batch culture mode as well as the enrichment of the medium with 0.1% casamino acids and 0.01 M EDTA were carried out. The optimal strategy (medium replacement, medium enrichment with 0.1% casamino acids and 0.01 M EDTA, and a methanol feeding strategy consisting of a continuous linear step increase—0.4 mL/L/h—of methanol flow rate from 0.8 to 2.45 mL/L/h) resulted in a production level of 600 mg/L and volumetric productivity of 120.0 mg/L/day, achieved at day five post-induction and additionally kept residual methanol levels below 2 g/L [72].

A signal sequence is usually fused to the *N*-terminal of the heterologous gene to direct the protein to the *P. pastoris* secretory pathway, being the α-mating factor prepro sequence from *S. cerevisiae* generally used. The full sequence of the α-mating factor has two protease cleavage sites for KEX2 and STE13, allowing the removal of extra amino acids by endogenous enzymes to yield the mature protein. The Srivastava research group [75] evaluated the effect of different secretion signals on the extracellular production of IFNα-2b in *P. pastoris* GS115 under the control of the AOX1 promoter. The authors constructed three different expression vectors—namely, pPIC9HSS with native secretion signal, pPIC9IFN with full *S. cerevisiae* α prepro signal sequence, and pPIC9αIFN with mutated α prepro signal sequence. Two immunoreactive bands corresponding to the target protein were observed with the recombinant strain containing pPIC9IFN; as this construct is based on the full α prepro signal sequence, the *ste13* gene becomes limiting and EA (glutamate-alanine) repeats are not completely removed from the mature protein, thus leading to the production of a mixture of mature proteins with and without extra amino acids in the *N*-terminal, easily distinguishable by electrophoresis [75]. However, a single band corresponding to IFNα-2b was observed in the supernatant of *P. pastoris* strain transformed with pPIC9αIFN (secretion level = 200.0 mg IFNα-2b/L). Matrix-Assisted Laser Desorption/Ionization- Time Of Flight (MALDI-TOF) analysis revealed that this protein is correctly processed by *P. pastoris* intracellular machinery and presents its native N-terminal, being additionally observed that it is recovered in a biologically active form. This work points out the importance of using suitable secretion signals to obtain correctly processed and mature IFN molecules in *P. pastoris* culture medium—namely, in what regards to the use of a mutated α prepro signal sequence (absence of Glu-Ala repeats) allowing the limitations imposed by the low production of the *ste13* gene to be overcome [75]. Additionally, targeting IFNα-2b fused to the α mating factor sequence of *Saccharomyces cerevisiae*, Padmanabhan [76] cultivated *P. pastoris* GS115 in a bioreactor under the control of AOX promoter and observed that all clones secreted two forms of IFN, in accordance with the results from Srivastava research group [75]. To overcome this issue, the authors evaluated the effect of using a low induction temperature, as well as supplementing the culture medium with dimethylsulfoxide (DMSO). Although lowering the induction temperature had no effect on the expression of the high molecular weight and incorrectly processed IFN isoform, the addition of 10% DMSO, both in shake-flask and bioreactors, highly decreased their quantity to 2% of the main band of IFN [76]. The authors demonstrate that supplementation of culture medium with DMSO helps to increase the expression of rightly processed IFNα-2b in secreted methanol-induced *P. pastoris* cultures [76].

Considering that protein disulfide isomerase (PDI) is one endoplasmic reticulum (ER)-associated molecular chaperone that results in increased protein yield and assists protein folding in ER lumen [81], Dsilva and coworkers [77] investigated the effect of co-expressing the *pdi* gene along with a codon-optimized version of the gene of IFNγ in *P. pastoris* GS115. To accomplish this aim, three plasmids were constructed: pPICZαA-IFNγ (pPICZαA carrying a copy of mature human IFNγ gene), pPIC9K-PDI (pPIC9K based vector, carrying a copy of *pdi* gene), and pPICZαA-IFNγ^opt^ (pPICZαA based vector, carrying a copy of codon-optimized human IFNγ gene). The transformants were inoculated into BMGY medium, then the yeast pellets were cultured in BMMH (0.5% methanol, 1.34% YNB (w/o amino acids), 4 × 10^−5^% biotin, 0.04% histidine, and 0.1 M potassium phosphate, pH 6.0) and 1% methanol was added every 24 h. The authors found that the expression of IFNγ was enhanced by 2.67-fold by co-expression of the *pdi* gene along with the target gene. However, the highest IFNγ production levels were achieved using GS115-IFNγ^opt^, in which the gene sequence was codon-optimized to *P. pastoris*. In the same study, the authors evaluated the effect of non-nutritional factors such as temperature (20, 25, 28, and 37 °C), pH (5–8), methanol concentration (0.25, 0.5, 1 and 2%), inoculum size (0.5, 1, 2, and 5%), and agitation rate (100, 175, 200, and 250 rpm) on the production yield of IFNγ from GS115-IFNγ^opt^ clone. While one factor was assessed, all other parameters were kept constant—namely, temperature of 28 °C, pH 6, agitation at 250 rpm, 2% inoculum size, and 0.5% methanol concentration. The results revealed that approximately 2.50 mg/L of IFNγ was produced at 25 °C, whereas the production level was decreased to 1.12 mg/L at 37 °C [43]. Concerning pH, the maximum production of IFNγ was found to be 2.00 mg/L at pH 7, and it decreased both at pH 5 and 8. For methanol concentration, the maximum production of IFNγ was 2.50 mg/L at 1% methanol and decreased at 0.25 and 0.5% of methanol, which may be attributed to limited carbon source and suboptimal level for transcription. Since *P. pastoris* grows in high cell density, more agitation is required to meet the oxygen demand. Thus, the maximum production of 2.30 mg/L of IFNγ was achieved at 250 rpm; once rpm was lowered, decreased IFNγ levels were observed. Finally, at 2% inoculum size, a maximum of 2.10 mg/L of IFNγ was produced [77]. Overall, this study [77] highlights the potential of three different strategies that can act independently or be combined to increase IFNγ yield in *P. pastoris*: co-expression of *pdi* gene and removal of codon bias through codon optimization may overcome protein misfolding and improve translational efficiency toward more stable mRNAs, whereas careful optimization of cultivation parameters provides an additional increase in IFN yield.

In 2019, Sivaprakasam and coauthors [78] expressed glycosylated IFNα-2b extracellularly under the control of the AOX1 promoter using Glycoswitch^®^ (BioGrammatics, Carlsbad, CA, USA). *P. pastoris* SuperMan5 (glycoengineered, protease deficient, and Mut^+^ strain). In this study, the authors used the design of experiments and artificial intelligence to investigate the effect of medium components such as glycerol, ammonium sulfate, and methanol on IFNα-2b production. Both glycerol and methanol play a major role in central carbon and energy metabolism and in protein production in *P. pastoris* [82]. Ammonium sulfate regulates the expression of genes involved in methanol utilization (AOX, PpDHAS, PpDAK) and peroxisomal genes (PpPEX1, 5, 8, and 14) at the transcriptional level [83]. The IFNα-2b production decreased with an increase in the concentrations of glycerol, ammonium sulfate, and methanol, due to the inhibitory effect of glycerol on *P. pastoris* growth and protein production, cell toxicity at higher methanol concentration resulting from oxidative stress, and formaldehyde accumulation. The optimal levels of the three medium components were found to be 46.06 g/L glycerol, 10.15 g/L ammonium sulfate, and 1.38% (**v/v**) methanol. Using these conditions in a bioreactor and in batch mode, the maximum production of total IFNα-2b was 436 mg/L, in which glycosylated and unglycosylated IFNα-2b corresponded, respectively, to 262 mg/L and 176 mg/L [78].

Ljubijanki and coworkers [79] successfully produced partially *N*-glycosylated IFN*β-*1 using *P. pastoris* GS115. The expression of the human gene was placed under the control of the *P. pastoris* AOX1 promoter, and the *S. cerevisiae* α-factor prepro-leader sequence targeted the protein to the secretory pathway. Four integrative plasmids (pPIC9IFN, pPIC9-IFNΔE-Y, pPIC9-EKRIFN, and pPIC9-EKREAEAIFN) containing different spacer peptides with varying amino acids compositions in sequence directly preceding the mature interferon sequence were constructed, envisaging to maximize interferon secretion, increase the KEX2 endopeptidase processing efficiency, and enable the authentic N-terminal sequence of natural IFNβ-1 to be formed. The protein expression reached yields between 6.000 and 12.00 g/L [79]. In general, the presence of Glu-Ala dipeptides in construct pPIC9-EKREAEAIFN increased the processing efficiency by KEX2 protease and the yield of secreted biologically active IFN, while 75% IFN was *N*-glycosylated [79]. Lastly, for the production of IFNλ, cDNAs encoding amino acids 23–200 or 20–200 of human IFNλ were cloned and joined to the sequence encoding the leader region (prepro segment) of the precursor of *S. cerevisiae* α-factor by Huang and coworkers [69]. The two-hybrid genes were subcloned into the yeast integrative vector pAO815 separately to construct expression plasmids bearing four tandem copies of IFNλ expression cassettes. Then, the plasmids were used to transform into *P. pastoris* GS115 strain, resulting in recombinant strains GS115/IFNλP 1P and GS115/IFNλG 1G with Mut^+^ or Mut^s^ phenotype. IFNλ was secreted into the BMM medium upon methanol induction, under agitation and controlled temperature of 30 °C [69]. However, the authors found that with the GS115/IFNλP 1P strain, KEX2 cleavage for mature IFNλ generation was inhibited by a proline at P’1. On the other hand, the recombinant strain GS115/IFNλ 1G secreted two forms of mature IFNλ with the same *N*-terminal sequence but with different molecular weights (22 and 35 kDa). Periodic acid–Schiff (PAS) staining revealed that although both proteins were glycosylated, the 35 kDa protein displayed higher affinity to PAS, being thus hyperglycosylated. The yield of the low molecular weight variant in GS115/ IFNλ 1G Mut^+^ was 65.0 mg/L in shake-flask, representing 57% of total secreted proteins [69].

As described below in Section 3.3.2, modifications can be introduced in IFN molecules to improve their pharmacological profile. To avoid complex chemical modification procedures, researchers often proceed to recombinant DNA technology to pursue the same goal. In this way Zhou and coworkers [80] reported the production of rHSA/IFNα-2b, a recombinant fusion protein composed of human serum albumin (HSA) genetically fused at its *C*-terminus to the *N*-terminus of IFNα-2b, separated by the protein linker Gly-Gly-Gly-Gly-Ser. rHSA/IFNα-2b was expressed in a Biostat C 15 L fermenter using *P. pastoris* transformed with pPIC9 vector and HSA natural signal peptide (instead of the original α factor signal peptide) to direct secretion of rHSA/IFNα-2b. After growing during 2 days at 30 °C, rHSA/IFNα-2b production was induced by methanol for about 50 h, and a concentration of 250 mg/L was obtained. In vivo studies carried out in monkeys show that the modified IFNα-2b exhibited an improved biological activity over IFNα [80].

**Figure 4:**
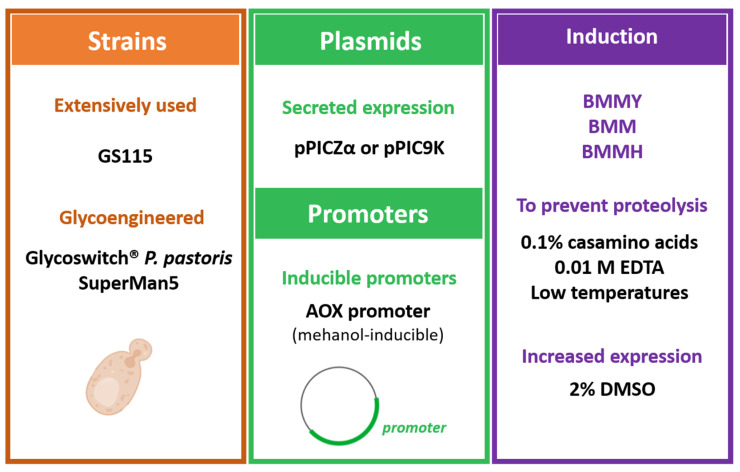
Optimized factors towards enhanced expression of IFNs using recombinant *P. pastoris*.

Overall, the studies analyzed in this sub-section demonstrate the enhanced ability of *P. pastoris* for the secretion of IFN molecules, although optimization of operational parameters is required to take full advantage of this system. The optimized parameters hitherto are schematized in Figure 4. Aiming to avoid extracellular proteolysis while increasing the yield of IFN, strategies have been designed, such as lowering the culture temperature and supplementing the medium with specific components, e.g., casamino acids. The α-mating factor is usually used as a signal sequence, which, however, may be incompletely processed, leading to different IFN isoforms with distinct molecular weights. This can be overcome by using mutated forms of the α-mating factor and by supplementing the culture medium with specific components such as DMSO. Also, at the genetic level, co-expression of molecular chaperones and the removal of codon bias may prevent protein misfolding and improve translational efficiency, which when coupled to the careful optimization of culture conditions increase the yield of secreted IFNs in *P. pastoris*. Some studies recall our attention to the glycosylation pattern of heterologous IFNs biosynthesized in *P. pastoris*, which may be hyperglycosylated. However, the half-life of IFNα2 may increase by hyperglycosylation [43]. Finally, it has been shown that recombinant IFN fusions, particularly with HSA, can be obtained by engineering the heterologous gene instead of performing chemical modifications at the end of the process.

### 3.2. Downstream Processing of Interferons

After production, the *downstream* processing is an extremely crucial stage that allows the extraction of the target protein from the harvested cells/culture supernatant, followed by its economical and efficient purification [4]. It should be remarked that the final level of purification and yield depends not only on the purification strategy but also on the upstream stage, since it influences the initial concentration of the protein and its purity [4,61,84]. The ideal purification process should be reliable, robust, feasible to apply at an industrial scale, fast, and cost-effective and should allow target products with high yield and purity to be obtained [4].

The downstream process aims to remove impurities while maintaining the chemical structure and biological activity of the target molecule and includes the following steps: (i) extraction/isolation, comprising the initial recovery of the product; (ii) purification–capture and intermediate purification by the removal of contaminants from the recovered product, and; (iii) polishing, removing contaminants and unwanted forms of the biomolecule of interest formed during the previous steps of the downstream processing [40,85].

#### 3.2.1. Cell Lysis and Interferon Recovery

**Figure 5:**
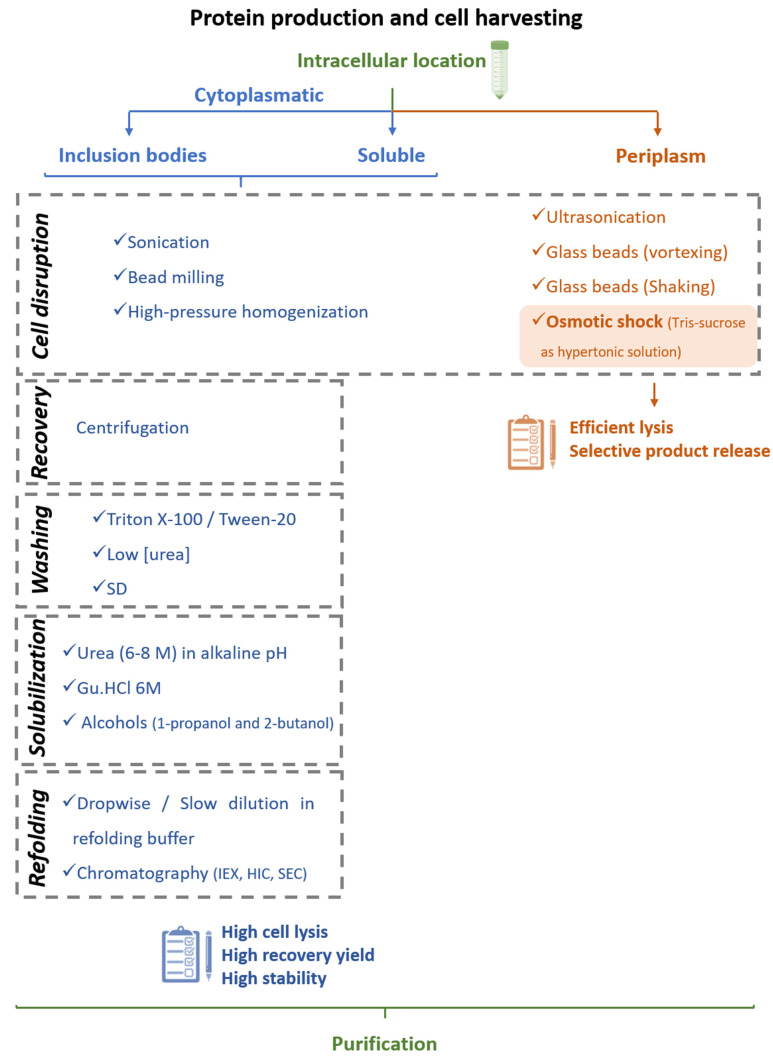
Primary recovery strategies for *E. coli* derived IFNs.

After the production and harvesting of fermentation broth or cell culture supernatant, cells have to be disrupted, extracted, or simply removed as the first step of product isolation [61]. This initial recovery/extraction stage depends on the physicochemical properties of the protein, the expression host, and the chosen production pathway [4,60]. Herein, we briefly address current strategies applied for the recovery of IFNs from *E. coli* and *P. pastoris* at a laboratory scale, strategies that may differ from those applied in industry. IFNs recombinantly produced in *E. coli* bacteria (see Section 3.1.1) can be obtained by resorting to two main routes, the periplasmatic pathway and the cytoplasmic pathway, and in this last case, IFNs can be obtained in a soluble form or as inclusion bodies. As a result, different recovery protocols must be implemented, as shown in Figure 5.

In cases where IFN is transported to the periplasm, selective disruption of the outer membrane is crucial to avoid complete lysis, thus ensuring that the target protein is recovered in a more purified form without soluble cytosolic proteins. Ariff and coworkers [86] compared the performance of ultrasonication, glass beads vortexing, and glass beads shaking for IFNα-2b from *E. coli* periplasm. The authors claimed that the amount of IFNα-2b obtained from the three protocols was comparable, respectively, 0.240, 0.172, and 0.201 mg/L for ultrasonication, glass beads vortexing, and glass beads shaking. Still, the selective product release (mg IFNα-2b/mg total protein) was, respectively, 65 × 10^−6^, 78 × 10^−6^, and 67 × 10^−6^ mg/mg [86]. Unlike mechanical methods-e.g., high-pressure homogenizer, hydrodynamic cavitation, and bead mill-which are usually considered not selective for the release of individual periplasmic proteins [87], the osmotic shock method (physical method) is referred to as a method with high selectivity [61,87]. This method involves the incubation of recombinant cells in a medium with high osmolarity (hypertonic, such as sucrose), followed by a dramatic change in osmolarity (hypotonic, such as water). Due to the rapid change in tonicity, the cell wall breaks, and proteins are released into the solution [87]. Through the investigation of different process parameters, Ariff and coworkers [87] observed that optimum release of IFNα-2b from *E. coli* periplasm was achieved with a cell concentration of 0.05 g/mL in a hypertonic solution (18% sucrose, 100 mM Tris, 2.5 mM EDTA—pH 8 and 25 °C) and 0.2 g/mL in a hypotonic solution (cold water –4 °C). Using these conditions and a sample volume of 1 mL, selective IFNα-2b release was determined to be 344.6 × 10^−6^ mg IFNα-2b/mg total protein [87], thus demonstrating a superior performance over mechanical methods [86]. Using the Tris-sucrose-dithiothreitol hypertonic buffer, Rodríguez and coworkers [54] additionally showed the improved performance of osmotic shock for selective release of periplasmic IFNγ over methods based on lysozyme, pipetting, or dilution.

In addition to the periplasm, recombinant IFNs can accumulate intracellularly in *E. coli* cytosol. As depicted in Figure 5, a cell disruption step is initially applied to recover intracellular proteins, after which four additional steps are required to recover proteins from inclusion bodies-namely, recovery, washing, solubilization, and refolding [61]. The cell disruption stage should maximize cell lysis, recovery yield, and stability of the recombinant protein. Moreover, protein oxidation and unwanted proteolysis should be minimized, being achieved, respectively, by the addition of reducing agents or protease inhibitors [88]. A wide range of techniques have been successfully applied to this end, including sonication [56,57,59], bead milling [60], and high-pressure homogenization [46]-this last technique also being suitable at an industrial scale [61].

**Figure 6:**
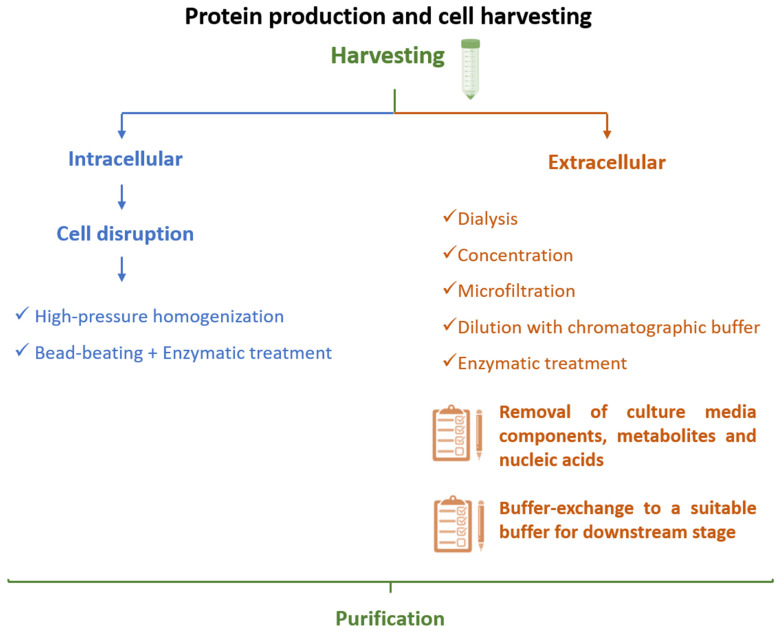
Primary recovery strategies for *P. pastoris* derived IFNs.

After cell lysis, inclusion bodies are isolated from soluble proteins by centrifugation [60] and are then subjected to different washing steps, e.g., with detergents (Tween 20 [89] or triton X-100 [60]), low concentrations of urea [60,90], or sodium deoxycholate [91]. Subsequently, solubilization occurs in a high concentration of denaturing agents such as urea (6–8 M) [92] or guanidine hydrochloride (6 M) [60,90] and can be enhanced at alkaline pH [5,49]. Milder solubilization methods employing 1-propanol or 2-butanol in the presence of sodium dodecyl sulfate (SDS) have also been demonstrated to be effective for IFNβ-1b solubilization [93]. During this process, dithiothreitol (DTT) is commonly added to decrease non-native disulfide bonds [60,94]. Finally, after solubilization, the inclusion bodies undergo the refolding process, in which various factors should be considered-namely, protein concentration, co-aggregation of protein contaminants, temperature, pH, and ionic strength. Protein refolding has been achieved by dropwise addition of denatured and solubilized protein to refolding buffer (containing reducing agents, reduced and oxidized glutathione, detergents, sugars, and amino acids, among other refolding additives) [49,59] or by slow dilution [46]. Chromatographic techniques involving ion-exchange (IEX) [92], size exclusion chromatography (SEC) [95], or hydrophobic interaction chromatography (HIC) [96,97], all relying on a gradual decrease of the concentration of denaturant, have also been applied for protein refolding. Using affinity chromatography and SEC, Norouzian and coauthors [98] studied the influence of pH (7 to 8.5) on the refolding efficacy and biological activity of IFNα-2b. From pH 7 to pH 8, the authors found that the refolding efficacy increased from 42.28% to 61.80%, and the comparative potency (biological activity determined as the inhibitory activity of IFN on the cytopathic effect of encephalomyocarditis virus on Hep2c cells) increased 1.48 times, highlighting the beneficial effect of adjusting pH during refolding to obtain highly bioactive IFN molecules [98]. Intracellular proteins are recovered from *P. pastoris* after a suitable cell lysis step, usually resorting to high-pressure homogenization [61], or (glass) bead-beating, which can be combined with enzymatic (zymolyase) treatments [70]. If IFNs are secreted to the culture medium, distinct strategies can be applied in the initial recovery step, including dialysis (sometimes followed by a concentration step using commercial devices), simple dilution with chromatographic binding buffer, or clarification based on microfiltration, as overviewed in Figure 6.

Dialysis allows the removal of culture media components as well as metabolites derived from the production stage and simultaneously permits buffer exchange to a suitable buffer compatible with the subsequent downstream techniques. In this way, distinct works have been performed using a wide range of buffers, e.g., 10 mM Tris NaCl 150 mM [75], water and then 20 mM NaH_2_PO_4_ 500 mM NaCl pH 7.4 [79], or cold sodium acetate buffer pH 4.5 with 2.5% sucrose, 0.2% Tween 80, and 0.5 mM EDTA [76], in which the target IFN is then subjected to chromatographic purification, respectively, SEC, immobilized-metal affinity chromatography (IMAC), and IEX. Dsilva and coworkers [77] concentrated the culture supernatant 100-fold before proceeding to purification. On the other hand, simple dilution with chromatographic binding buffer of culture supernatant before chromatography has also been performed [69,74,80], allowing the concentration of possible interferents in the supernatant to decrease and in which protein concentration is achieved after purification (addressed in more detail in Section 3.2.2). Kallel and coworkers [72] used microfiltration through the application of 0.1 hollow fiber cartridge and found that almost 95% of IFNα-2b was retained within the retentate. The authors additionally observed that addition of triton X-100 or NaCl to the culture medium before microfiltration improved the recovery yield of this step [72].

In sum, the recovery/extraction stage depends, among other aspects, on the chosen production route [4,61,62]. In the case of IFN, the cytoplasmic pathway using inclusion bodies is the most widely used, presenting a higher production yield when compared with the periplasmatic pathway. Besides, due to a process of cell lysis, solubilization, and optimized refolding, it is possible to minimize the disadvantages associated with this type of protein aggregate, increasing the IFN recovery yield to promising levels in the end [60].

#### 3.2.2. Chromatography-Based Purification

**Figure 7:**
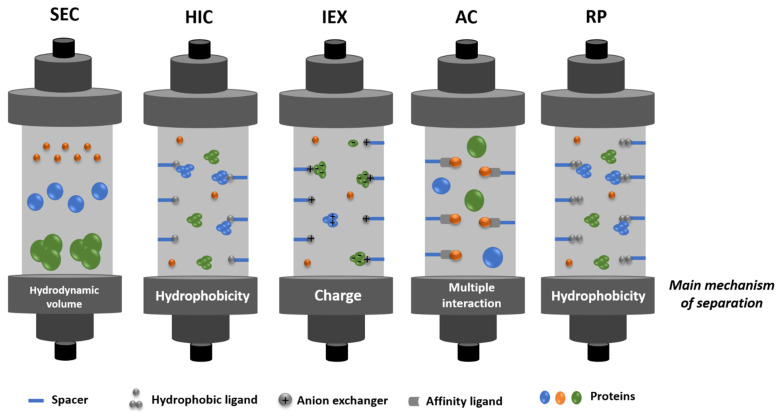
Separation principles of distinct types of chromatographic techniques applied for the isolation and purification of IFNs (SEC–Size exclusion chromatography, HIC–Hydrophobic interaction chromatography, IEX–Ion-exchange chromatography, AC–Affinity chromatography, RP–Reverse phase chromatography).

Therapeutic IFNs, as with other biopharmaceuticals, must be obtained with high purity in the absence of host cell proteins, endotoxins, or contaminants. Despite the increasing competition from non-chromatographic techniques and pressure to reduce costs and increase throughput, packed-bed chromatography is still the dominant technique applied for biopharmaceuticals purification [99]. Current chromatographic methods generally applied to the isolation and purification of IFNs include (i) affinity chromatography, (ii) IEX, (iii) SEC, (iv) reverse-phase, and (v) HIC. Representative studies of the chromatographic purification of IFNs are overviewed in Table 5, and the separation principles of each method are schematized in Figure 7.

**Table 5:** Representative studies of the application of chromatographic techniques for the purification of distinct classes of recombinant therapeutic IFNs.

Chromatography	Column	IFN	Host	IFN Concentration	Recovery Yield (%)	Purity (%)	Specific Activity (IU/mg)
IMAC [77]	His-Trap FF affinity column with Ni^2+^	8xHis IFNγ 8x His IFNγ-PDI 8x His IFNγ^opt^	*P. pastoris* GS115	0.009 mg/L	36.00	56.25	Not reported
				0.030 mg/L	54.54	63.83	
				0.120 mg/L	52.17	80.00	
IMAC [59]	His Bind Quick 900	10xHis IFNα-2	*E. coli* BL21 (DE3)-RIL	21.0 mg/L	16.00	18.00	1.8 × 10^8^
		10xHis IFNα-8		55.0 mg/L	44.00	44.00	3.4 × 10^8^
		10xHis IFNα-828		30.0 mg/L	26.00	24.00	7.5 × 10^8^
IMAC + SEC [56]	GSTrap Fast Flow + Sephacryl S-100	GST-IFNα-2	*E. coli* BL21 *E. coli* Origami B	100 mg/L	NR	NR	2.0 × 10^8^
IMAC [79]	Hi-Trap FF affinity column with Cu^2+^	IFNβ-1	*P. pastoris* GS115	10.0 mg/L	NR	80.00	2–3 × 10^7^
IMAC + SEC [100]	His-Trap FF affinity column with Ni^2+^	IFNα-2 Thymosin α1	*E. coli* BL21 (DE3)	950 mg/L	69.00	98.00	Biologically active (Not comparable)
IMAC + AEX [51]	His-Trap FF affinity column + HiTrap Q HP	MBP-IFNα-2b	*E. coli* BL21 (DE3)	14.4 mg/L	10.50	99.80	Biologically active (Not comparable)
IAC [101]	IFNα-2a antibody conjugated to Sepharose 4B	GFE-IFNα-2a	*E. coli* BL21 (DE3)	1,05x10^3^ mg/L	0.520	>95.00	2.5 × 10^8^
AC [91]	Blue-Sepharose Fast Flow	IFNβ	*E. coli* BL21 (K12)	NR	NR	93.50	Biologically active (Not comparable)
AEX [49]	Q Sepharose Fast Flow	IFNα-2b	*E. coli* DH5α	3.00x10^3^ mg/L	58.00	99.00	3 × 10^9^
AEX [76]	Q Sepharose Fast Flow	IFNα-2b	*P. pastoris* GS115	900 mg/L	93.00	90.00	>2 × 10^8^
CEX [92]	SP-Sepharose Fast Flow	IFNγ	*E. coli*	100 mg/L	54.00	95.00	7.5 × 10^5^
CEX [102]	SP Sepharose XL	IFNγ	*P. pastoris* X-33	135.2 mg/L	56.00	90.00	1–1.4 × 10^7^
CEX + SEC [72]	Sepharose SP + Sephacryl S100	IFNα-2b	*P. pastoris*	183 mg/L	30.00	100.0	1.5 × 10^8^
CEX + SEC [69]	SP Sepharose Fast Flow + Superdex 75	IFNλ-1	*P. pastoris* GS115	NR	NR	>98.00	NR
AEX + SEC [74]	Q Sepharose Fast Flow + Superdex 75	IFNα-2b	*P. pastoris*	298 mg/L	64.00	>95.00	1.9 × 10^9^
RP [45]	C18	IFNε	*E. coli* DH5α	800 mg/L	NR	NR	6 × 10^5^
IMAC + RP [57]	His-Trap FF affinity column with Ni^2+^+ C8	SUMO-IFNcon	*E. coli* SHuffle™	50.0 mg/L	NR	98.00	960 × 10^6^
AEX + CEX [90]	Q Sepharose Fast Flow + SP-Sepharose Fast Flow	NGR-IFNα-2a	*E. coli* BL21 (DE3)	18.0 mg/L	NR	>98.00	6.2 × 10^8^
AC + HIC + AEX + SEC [80]	Blue Sepharose Fast Flow + Phenyl Sepharose HP + Q Sepharose Fast Flow + Sephadex G25	HSA-IFNα-2b	*P. pastoris*	64.0 mg/L	25.40	97.00	6.3 × 10^5^
CEX + AC + SEC [103]	SP Sepharose Fast Flow + Blue Sepharose 6 Fast Flow + Sepharyl S-100	IFNλ-1	*CHO cells*	NR	NR	90.00	1 × 10^6^
SEC [104]	Sephacryl S-200	IFNα-2a	*E. coli* BL21 (DE3)	NR	82.00	92.00	1.2 × 10^8^
SEC [95]	Superdex 75	IFNγ	*E. coli* DH5α	NR	67.10	NR	1.2 × 10^7^

Abbreviations: AC–Affinity chromatography; AEX–Anion-exchange chromatography; CEX–Cation-exchange chromatography; HIC–Hydrophobic interaction chromatography; IAC–Immunoaffinity chromatography; IMAC–Immobilized metal-affinity chromatography; NR–Not reported; RP–Reverse-phase chromatography; SEC–Size exclusion chromatography.

Affinity chromatography allows a specific type of protein to be isolated from a mixture of proteins contaminants and is based on the affinity of proteins to specific ligands, for instance, metal cations or antibodies, respectively, in IMAC or immunoaffinity chromatography [40]. Recombinant proteins produced with attached amino acid sequences as fusion partners-“*tags*”-(GST, maltose-binding domain, hexahistidine–6xHis, among others) exhibit high specificity and affinity to chromatographic resins [39,51,56]. In IMAC, proteins containing at least six histidines (as a *N*- or *C*-terminal fusion partner) exhibit high affinity to Ni^2+^, Cu^2+^, or Zn^2+^, immobilized in a matrix containing a metal chelating group [84]. After purification, the tag can be removed by proteases, and additional purification steps are often required to purify the target protein [84]. Dsilva and coauthors [77] used the Ni-NTA (nitrilotriacetic acid) IMAC column for the purification of 8xHis-tagged extracellular recombinant IFNγ from *P. pastoris* GS115 crude broth. The authors found that the purity of IFNγ was positively correlated with the production levels: the codon-optimized version of IFNγ-GS-IFNγ^opt^-, the IFNγ co-expressed with PDI-GS-IFNγ-PDI- and the native version, GS-IFNγ-were obtained, respectively, with purities of 80%, 63.83%, and 56.5% (Table 5) [77]. This study reinforces the premise defended by several researchers in which the purification yield seems to be proportional to the initial concentration of IFN and its purity [4]. Indeed, higher purification yields were achieved when the initial concentration and purity of IFN was higher [77], again remarking on the importance of optimizing the upstream stage in the global process of biopharmaceuticals manufacturing. Moreover, the recovery of IFNγ was almost similar for GS-IFNγ^opt^ (52.17%) and GS-IFNγ-PDI (54.54%) [77]. Platis and Foster [59] successfully reported the purification of 10xHis-tagged (placed at the *N*-terminus) IFNα (IFNα-2 and IFNα-8 and their hybrids) from *E. coli* BL21(DE3)-RIL inclusion bodies. The final concentration of purified IFNs ranged from 5.00 to 15.0 mg/L of culture, with purification ratios (%) for IFNα-2 and IFNα-8 of 18% and 44%, respectively [59]. The authors hypothesized that the variation in the purification ratio could be due to stereochemical differences among the different constructs and that both yield and purity could be improved with additional optimizations (e.g., using higher capacity cartridges and by adjusting the initial amount of IFNs) [59]. Recently, Aslam and coauthors [100] reported the production and respective purification of IFNα-2-thymosin α1 fusion protein (IFNα-2 in combination with thymosin α1) in *E. coli.* IFNα-2-thymosin α1 fusion protein was expressed with 6xHis-tag at *C*-terminus. Through Ni-affinity chromatography, a purity of approximately 98% and a final yield of 69% (950 mg/L of cell culture) were obtained. Regarding biological activity, and although there is no mention to specific activity in quantitative terms, it was found that IFNα-2-thymosin α1 provided an increase in the level of expression of the caspase-3, BAX, and p53 and a decrease in the *VEGF* and *Bcl-2* mRNA, in comparison with standard IFNα-2 [100]. In sum, due to the observed synergistic effect, IFNα-2-thymosin α1 fusion protein showed significantly higher anticancer activity in comparison with the individual contribution of both polypeptidic chains [100].

Although affinity chromatography has been considered a fast purification method with high resolution, it should be mentioned that in some cases it is necessary to remove the fusion tags, as well as to verify the absence of divalent metals in the final sample [84]. Fathallah and coworkers [56] demonstrated that despite the fusion of IFNα-2b to GST improved the solubility of the target protein, removal of the fusion tag was not optimal, even with different enzymatic concentrations. After engineering the GST–IFN junction that included the thrombin cleavage site (deletion of three amino acids and removal of codon bias of glycine at position–5), thrombin cleavage was highly improved, as monitored by electrophoresis. Protein purification was achieved using GSTrap FF affinity column, followed by a thrombin incubation step and final size-exclusion purification to remove glutathione and thrombin. The authors reported a final yield of pure IFNα-2b of 100 mg/L [56]. A study by Laurine and coworkers [57] allowed approximately 50.0 mg/L *E. coli* culture of recombinant SUMO-IFN-consensus of at least 98% purity to be obtained, evaluated by RP high-pressure liquid chromatography. Since the SUMO protein contains an *N*-terminal his-tag, the target protein was first purified using IMAC to isolate the SUMO-IFN-con fusion protein from the soluble fraction. Cleavage of the purified SUMO-IFN-con was conducted using 1 unit of SUMO protease to digest 10 µg of SUMO-IFN-con protein and allowed digestion of 98% of the fused protein [57]. An IMAC purification step was then applied to isolate the IFN-con from the His-tag-containing undigested fusion protein and the SUMO fusion partner. Using this method, the native IFN-con was collected in the flow-through, while the His-tagged SUMO fusion partner, the uncleaved SUMO-IFN-con, and the SUMO protease—all containing a His-tag—were retained on the column and eluted with imidazole. Notably, the released IFN-con was stable and present in a soluble form once the SUMO fusion partner was removed. Using an A549/EMCV antiviral assay, the specific activity of the recombinant IFN-con was determined to be 960 × 10^6^ IU/mg. Comparison of the antiviral activity of the produced IFN-con with IFNα-2a showed that IFN-con displayed 2.8 times higher activity, which was in good agreement with what has been reported in the literature for pure protein [57]. In addition to IMAC, other sub-types such as immunoaffinity chromatography have been applied for IFNs purification. Zhang and coauthors [101] purified the recombinant IFNα-2a-GFE fusion protein from *E. coli* cell lysates in one step by monoclonal antibody immunoaffinity chromatography using the resin Sepharose 4B conjugated with antihuman IFNα-2 monoclonal antibody. It was possible to obtain a purity >95%. Another main highlight of this article is the fact that the GFE protein coupled to IFNα-2a, originating GFE-IFNα-2a, has high selectivity for receptors located in the lungs and kidneys. In this way, GFE can be useful to deliver IFNα-2a to the mentioned organs, where IFN can then trigger its therapeutic actions. The therapeutic effect was not affected by the process of protein fusion, production, and purification, since the specific activity of GFE-IFNα-2a is very close to the standard value of IFNα-2a [101].

IEX provides high resolution under mild conditions and is based on a reversible interaction between surface charged groups from the protein and opposite charged groups in the matrix. [40,84]. A key consideration in IEX is the isoelectric point (pI) of the target protein and the pH of mobile phase buffers since the protein can present an overall positively or negatively charged surface. Indeed, depending on if the pH is below or above the pI, the target protein is positively or negatively charged, and the process is termed cation-exchange or anion-exchange chromatography [84].

In the study by Padmanabhan and coauthors [76], recombinant IFNα-2b from *P. pastoris* crude supernatants was efficiently purified (90%) in a single step using the anion-exchanger Q-Sepharose. Most of the impurities bound to the resin and the protein of interest were recovered in the flow-through (93% recovery). Additionally, it was reported that the target protein had a structural similarity of approximately 78% with alpha-class IFNs and a specific activity within the expected values, indicating that the structure and biological properties of IFNα-2b were maintained [76]. Srivastava and coauthors [49] also obtained high purification efficiencies (99% judged by silver-stained electrophoresis gels) with a final recovery of 58%. In this case, the dialyzed IFNα-2b was loaded on a Q-Sepharose column equilibrated with 50 mM Tris–HCl (pH 8.4) since the protein has an isoelectric point of 5.9. After washing, the bound protein was eluted with a linear salt gradient (0–1 M NaCl) in a high purity degree [49]. Cho and coauthors [92] used only one chromatographic step for the refolding and purification of IFNγ. The IFNγ was expressed in *E. coli* as inclusion bodies. Triton X-100 was initially used to wash the IFNγ inclusion bodies before chromatographic refolding. The refolding process was performed by gradually decreasing the concentration of urea in the column after the denatured IFNγ protein bound to the ion-exchange gel SP-Sepharose Fast Flow [92]. The refolding and purification process of denatured IFNγ was carried out simultaneously and the purity of refolded IFNγ was up to 95%. Cation exchange chromatography presents some advantages, including the ability to perform protein refolding at high protein concentration, in little time, and enables refolding and purification to be performed in one step. Therefore, it is considered a viable process for large-scale applications. Under the optimum conditions, the specific activity of IFNγ was up to 7.5 × 10^5^ IU/mg and active protein recovery was 54% [92]. Another work reported the purification of IFNγ from *P. pastoris* cultures. IFNγ was purified with cation exchange chromatography, where the concentrated sample was loaded onto SP Sepharose XL (considering the isoelectric point of approximately 8.1–9.1). It was possible to obtain 90% purity with 56% recovery [102].

SEC, also referred to as gel permeation chromatography or molecular sieving, permits the separation of proteins based on differences in their hydrodynamic volume (size and shape). Generally, this type of chromatography is suitable for the separation of proteins with considerable differences in their molecular weight, and it allows buffer-exchange or desalt to the desired buffer [40]. In addition to the application of SEC for the removal of fusion partners and proteases [56], Cho and coauthors [95] focused on gradient SEC for the refolding of IFNγ obtained from *E. coli* inclusion bodies. The inclusion bodies were first solubilized in 8 M urea as the denaturant, and the refolding process was then performed by decreasing the urea concentration on the SEC column (Superdex 75 gel medium) to suppress protein aggregation [95]. The combination of the buffer-exchange effect of SEC and a moderate urea concentration in the refolding buffer resulted in an efficient route for producing correctly folded IFNγ, with protein recovery of 67.1% and specific activity up to 1.2 × 10^7^ IU/mg. In another study [104], a decreasing urea gradient SEC for the refolding of recombinant IFNα-2a overexpressed as inclusion bodies in *E. coli* was investigated. In the chromatographic process, the denatured IFNα-2a passed along the 8.0–3.0 M urea gradient and refolded gradually [104]. Under the optimum conditions, 1.2 × 10^8^ IU/mg of specific activity, purity of 92% and mass recovery of 82% was obtained during this process. Urea gradient SEC is a high-efficiency method in terms of specific activity and mass recovery for refolding and purifying IFN in a single step. The main advantage related to these works is the decrease in the cost and time associated with refolding and (at least partial) purification, given that they are carried out simultaneously.

Reverse-phase chromatography separates proteins based on differences in their relative hydrophobicity, and since the concentration of hydrophobic ligands in the matrix is generally very high in comparison with HIC (addressed below), elution generally requires the use of organic solvents [40]. Hou and coworkers [45] applied reverse-phase high-pressure liquid chromatography (buffer A-0.1% trifluoroacetic acid and buffer B-99.9% acetonitrile and 0.1% trifluoroacetic acid) to purify recombinant IFNε and to facilitate refolding of the protein. Purified IFNε protein was obtained with a concentration of 8.00 mg/L *E. coli* culture. However, a functional study of IFNε demonstrated that its antiviral activity was about 60 times less potent than IFNα-2b (6 × 10^5^ IU/mg) [45]. By requiring an organic solvent as eluent, this technique is mostly used in analytical chromatography, since proteins can be recovered in a denatured form, which must not occur with protein-based biopharmaceuticals [40]

Similar to reverse-phase chromatography, protein hydrophobicity is the major factor governing the interaction of therapeutic proteins with hydrophobic ligands in HIC, in which high concentrations of salt (e.g., ammonium sulfate) are usually applied to expose the surface hydrophobic patches of proteins. However, elution is generally milder, being accomplished by a decreasing gradient of salt concentration [84] and thus favoring the recovery of target proteins with higher biological activities. Wu and coworkers [96] implemented a technology for renaturation and simultaneous purification of IFNγ from *E. coli* inclusion bodies using HIC. The general process comprises the injection of solubilized inclusion bodies (7.0 M GuHcl) with a specific buffer containing 3.0 M ammonium sulfate (buffer A), followed by gradient elution to buffer B (same as the binding buffer but without ammonium sulphate) during specific time periods. Silica-based HIC matrices with different end-groups (polyethylene glycol (PEG)-200, PEG-400, PEG-600, PEG-1000, furfural, pyridine, phenyl) were evaluated and found to have a more significant influence in refolding, contrary to the mobile phase composition (distinct salts were screened, including KH_2_PO_4_, NaCl, NaAc, Tris, among others). In general, the silica-based matrices with PEG-200, PEG-400, PEG-600, and PEG-1000 allowed the highest bioactivity recoveries; moreover, using a flow-rate of 100 mL/min and a gradient elution by one step in 4 h, the purity and bioactivity recovery approached 95% and 8.7 × 10^7^ IU/mg, respectively. This strategy allowed improvements at the level of purity, mass and bioactivity recoveries, cost, and time over a conventional dual-step strategy based on (i) the renaturation by dilution method and (ii) purification by several chromatographic techniques [96]. On the other hand, Su and coworkers [97] developed a dual-gradient strategy based on HIC involving a decrease in GuHCl concentration and an increase in PEG concentration, which allowed enhancements of the refolding yield of consensus IFN. The authors found that using conventional HIC media, gradient elution provides a gentle, smooth change of the solution environment that allows the denatured protein to refold gradually and that leads to the formation of the correct structure. In comparison with the dilution method, the use of PEG (molecular weight of approximately 200 g/mol) as an artificial chaperone has a more pronounced effect for on-column refolding, allowing approximately a 2.6-fold increase in specific activity and a 30% increase in soluble protein recovery [97].

Along with single-step chromatographic processes, several reports explore multiple chromatographic processes, envisaging to increase the purity of target IFNs. In 2016, Vu and coauthors [51] used IMAC and anion exchange chromatography for the purification of the MBP-IFNα-2b fusion protein. MBP-IFNα-2b was initially purified by IMAC and then the purified MBP-IFNα-2b fusion was subjected to Tobacco Etch Virus (TEV) protease digestion to remove the MBP tag—with a cleavage efficiency of approximately 89% [51]. Envisioning the removal of uncleaved fusion protein, cleaved MBP tag, and TEV protease, the resulting digestion products were then subjected to a second IMAC purification step; this was possible because fusion proteins and cleaved MBP tags have a 6xHis tag at the *N*-terminus while TEV contains a 6xHis tag at its *C*-terminus. The final anion exchange chromatography aided in reducing endotoxin levels (only 0.46 EU/μg of the final protein product) and the remaining impurities, allowing at the end a purity of 99.8% [51]. However, it is important to notice that the extraction yield (10.5%, corresponding to 14.40 mg IFNα-2b/L) in this study was calculated based on the biological activity and not on mass percentage. Therefore, this yield cannot be compared with the ones discussed before. Although its specific activity is not mentioned, the authors claim that the levels of endotoxins were quite low, which is an essential aspect for the application of IFNα-2b as a therapeutic drug [51]. Another report by Cheng and coworkers [74] disclosed the application of anion-exchange chromatography (Source Q ion exchange column) and SEC (Superdex 75) for the purification of IFNα-2b from *P. pastoris* culture medium. The purity of the recombinant protein was higher than 95%. The final recovery yield of the recombinant protein was 64%, which translates to 298.0 mg of the purified protein from 1 L of the supernatant, and its identity to IFNα-2b was confirmed by NH_2_-terminal amino acid sequence analysis. The bioassay of the recombinant protein gave a specific activity of 1.9 × 10^9^ IU/mg [74]. A two-step process involving cation exchange chromatography and SEC was explored by Huang and collaborators [69] to purify IFNλ from *P. pastoris* fermentation supernatant. Cation exchange chromatography was a crucial step to remove native secreted proteins of *P. pastoris*. The crudely purified proteins were further purified on a Superdex 75 size exclusion column. IFNλ was eluted with a purity of >98% [69].

Zheng and coauthors [103] reported the purification of IFNλ-1 through four purification steps: ammonium sulfate precipitation, cation exchange chromatography, affinity chromatography, and SEC (Sepharyl S-100 gel). The classic ammonium sulfate precipitation method was performed to isolate IFNλ-1 protein. In the second step, a fraction of acidic proteins were removed using cation exchange chromatography (SP Sepharose Fast Flow column), since the isoelectric point of IFNλ-1 is 8.1 [103]. Further purification was performed with Blue Sepharose 6 Fast Flow affinity chromatography; a purity of up to 58% was achieved because the gel has a high affinity for IFN, enabling the removal of some impurities. At the end, the application of SEC allowed recovering IFNλ-1 with a final purity of about 90%. The antiviral activity of IFNλ-1 was determined to be 1 × 10^6^ IU/mg using the vesicular stomatitis virus (WISH-VSV) assay system. According to the authors [103], this value was higher in relation to some biopharmaceuticals, based on this IFN, that were already in the biopharmaceutical market. Thus, although the purification process has several steps, the authors consider this strategy quite efficient in terms of purification, with potential clinical application due to the high biological activity of recovered IFN [103].

As described in detail below (Section 3.3.2), IFN fusion proteins can have several advantageous effects, such as improved biological activities or increased half-lives, thereby allowing a decrease in the frequency and dosage of administration and a reduction in the associated side effects. For instance, Zhang and coauthors [90] reported the expression and purification of an NGR-IFNα-2a fusion protein. The NGR (Asn-Gly-Arg) peptide is a tumor-homing peptide used to increase the antitumor activity of IFNα-2a and lower the dose. The fusion protein was expressed in *E. coli* inclusion bodies. After solubilization with 6.5 M guanidine hydrochloride, the sample was purified by IEX—namely, anion exchange chromatography and cation exchange chromatography. The final purity of NGR-IFNα-2a was more than 98%, and the final purification yield of NGR-IFNα-2a was approximately 18.0 mg/L. Finally, the purified protein reached a specific activity of 6.2 × 10^8^ IU/mg, demonstrating that the fusion partner did not interfere with folding or its ability to bind to IFNα-2a receptors. Additionally, it was reported that NGR-IFNα-2a had a stronger antitumor effect and a high selectivity to target tumor vessels in comparison with IFNα-2a, allowing a decrease in its dosage, providing advantages in combating cancer and in reducing side effects [90]. Zhou and coauthors [80] also reported the production of a fusion protein through coupling IFNα-2b to HSA-HSA-IFNα-2b. The fusion protein was purified using a total of four chromatographic processes: affinity chromatography (Blue Sepharose Fast Flow), HIC (Phenyl Sepharose), anion exchange chromatography (Q-Sepharose Fast Flow), and SEC (Sephadex G25). Blue Sepharose Fast Flow was used to capture protein from culture broth because the fusion protein contained albumin, which can specifically bind to Cibacron Blue. Fractions pooled from Blue Sepharose Fast Flow contained 2 mol/L sodium chloride, which is suitable for HIC at high conductivity. Then, HSA-IFNα-2b was eluted from the Phenyl Sepharose HP column by 10 mmol/L sodium phosphate and was applied to the Q Sepharose Fast Flow column directly. The purity of the prepared HSA-IFNα-2b was 97%, and about 64.0 mg HSA-IFNα-2b was purified from 1 L cell-free broth, i.e., about 25.4% recovery yield was obtained. However, the specific activity was lower in comparison with the standard IFNα-2b, 6.3 × 10^5^ IU/mg [80].

Overall, a wide range of chromatographic techniques for exploring different types of interactions between chromatographic ligands and target IFNs-ranging from ionic, hydrophobic, van der Waals or hydrogen-bonding—have been applied for the purification of IFNs with purities above 90%. However, it should be remarked that multiple steps of (chromatographic) purification are usually required to obtain higher purity values, leading to a decrease in the recovery yield and an increase in the overall cost and time of the process. This is indeed one of the main drivers toward the development of improved purification processes, mostly accomplished by the design of new ligands, resins, and by taking advantage of process modelling, operating, and control strategies.

Although the use of fusion partners such as 6xHis tags enables simple and facile purification using IMAC matrices for structural and functional studies, the yields may be far from the desired, highlighting the additional importance of the careful design of the protease cleavage site to ensure optimal cleavage. The large number of studies reporting the expression of IFNs as *E. coli* inclusion bodies, mostly in the first decade of this century, has led to the development of simultaneous refolding-purification procedures, which collectively represent a time- and cost-saving approach for obtaining high-purity and biologically active IFNs.

#### 3.2.3. Alternative Purification Strategies

**Figure 8:**
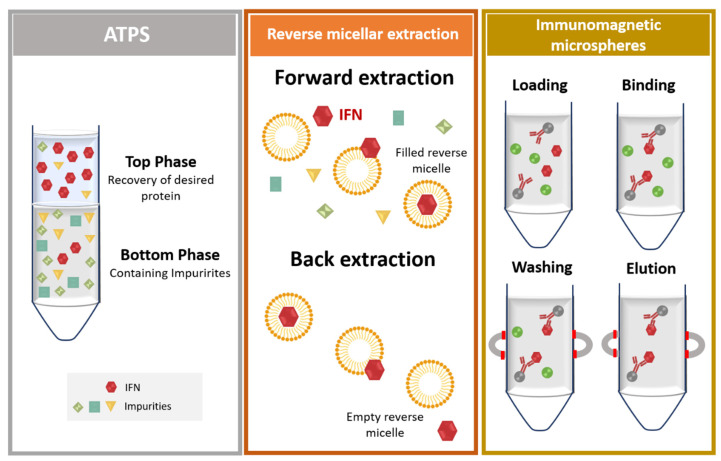
Separation principles of alternative techniques applied for the purification of IFNs (ATPS–Aqueous two-phase system).

Over the years, alternatives to the widely applied and effective column liquid chromatographic processes have been described, mostly aiming to overcome their high cost and limited capacity [85]. Consequently, new techniques have been suggested, such as aqueous two-phase systems (ATPS) [5,105,106], cationic surfactant-based reverse micellar extraction [107], and immunomagnetic microspheres [108], schematized in Figure 8. These techniques aim for a high recovery yield and purity through the least possible number of steps, easy application on an industrial scale, a process that is human- and environmentally friendly, and a system that is cost-effective [4,61,84]. If all of these conditions are obtained, access to different types of biopharmaceuticals based on IFNs will be facilitated, enabling better treatment for various pathologies.

ATPS, also known as aqueous biphasic systems (ABS), are a type of liquid–liquid extraction technique consisting of two immiscible water-rich phases that separate above given concentrations, one of the phases being enriched in one of the solutes while in the other phase the second component prevails [109]. In comparison with other extraction techniques, ATPS display several advantages, spanning from their environmentally friendly and biocompatible character (mostly due to the high water content in both phases), low cost, continuous operation, and ease in scaling-up [109,110]. A wide range of phase-forming components can be applied, such as polymers, salts, ionic liquids, surfactants, and alcohols, to upgrade their performance toward the extraction of therapeutic proteins with high purity and yield.

Ling and coauthors [105,106] investigated the use of ATPS for the purification of periplasmic IFNα-2b from *E. coli* rosetta-gami2 (DE3) cultures. In the first study [105], an ATPS composed of PEG and potassium phosphate was investigated, as well as the influence of system parameters, including PEG molecular weight (molecular weight–MW-approximately 6000, 8000, and 10,000 g/mol), tie-line length, volume ratio, crude stock loading, system pH, and NaCl concentration (%, *w/w*). The results showed that the optimum condition to obtain a high purification factor of IFNα-2b in a single step was achieved by a system composed of 4% (**w/w**) PEG 8000, 13% (*w/w*) potassium phosphate, 0.5% NaCl, and 10% (*w/w*) crude stock, all at pH 6.5. A recovery yield of 40.7% was obtained with the optimized ATPS [105]. A year later, the same authors reported the use of alcohol/salt ATPS for IFNα-2b purification [106]. The influence of nine biphasic systems comprising alcohol-based top phases (ethanol, 1-propanol, and 2-propanol) and salt-based bottom phases (ammonium sulfate, di-potassium hydrogen phosphate, and monosodium citrate) on IFNα-2b purification was studied. The results showed that the optimum condition for the purification of IFNα-2b was achieved in ATPS composed of 18% (*w/w*) 2-propanol with 22% (*w/w*) ammonium sulfate in the presence of 1% NaCl. A recovery yield of 74.64% was obtained from the optimized ATPS. In both studies, the IFNα-2b purification performance was evaluated by SDS-PAGE and provided as a purification factor. A purification factor of 26.30 was obtained with polymer/salt ATPS [105], while with alcohol/salt ATPS [106] the purification factor was lower (16.24), meaning that the latest system was less efficient for the purification of IFNα-2b. Although the specific activity of the IFNα-2b was not reported and its conformational stability not studied, these results suggest that polymer/salt and alcohol/salt ATPS are a valuable alternative for IFNα-2b extraction and purification since they represent simpler, cheaper, and faster one-step purification methods. More recently, Pedro and coworkers [5] investigated the application of ILs as adjuvants (at 5 wt%) in ATPS constituted by PEG (MW approximately 600 g/mol) and polypropylene glycol (MW approximately 400 g/mol) (PPG400) at constant pH (8) to purify recombinant IFNα-2b from *E. coli* BL21 inclusion bodies. ILs are liquid molten salts composed of large and unsymmetrical organic cations and organic or inorganic anions. Due to the high number of ion combinations and respective chemical structures, they present a tunable character that allows researchers to adjust their physicochemical properties to meet the requirements of specific applications [109]. It was observed that IFNα-2b tends to migrate to the PEG-rich phase (being the phase also enriched in IL), whereas the remaining proteins tend to precipitate at the interface (fitting within the three-phase partitioning approach). In comparison with the ATPS without adjuvant, most systems comprising ILs as adjuvants lead to enhancements in the purification factors of IFNα-2b—namely, from 2.28 up to 6.77—with extraction efficiencies above 90% [5]. The purity of IFNα-2b was found to be maximized using ILs composed of aromatic cations and anions with high hydrogen-bond basicity (1-butyl-3-methylimidazolium acetate, [C_4_mim][CH_3_COO]), and the secondary structure of the target protein was found to be preserved during the purification step. Overall, this study demonstrated the ability of ILs to tune the characteristics of the ATPS coexisting phases toward improved purification processes by taking advantage of the designer solvent ability of ILs [5]. In summary, the described results indicate that optimized alternative purification platforms such as ATPS represent a promising technique for the recovery and purification of IFN.

Dasu and coworkers [107] reported the single-step purification of IFNγ from the fermentation broth of *Kluyveromyces lactis* using cationic surfactant-based reverse micellar extraction. This technique involves the solubilization of biomolecules in the water pool of reverse micelles, which are nanometer-sized water droplets contained inside a boundary created by surfactant molecules. Protein extraction using reverse micelles can be divided into two steps: (i) forward extraction, where the target protein present in the fermentation broth is transferred to the water pool of reverse micelles; and (ii) back extraction, where the target protein is released from reverse micelles to a fresh aqueous phase. After optimization, forward extraction efficiencies of 78, 93, and 98% were obtained, respectively, using aqueous phase pH 12, 150 mM cetyltrimethylammonium bromide (CTAB), and 0.2 M NaCl. On the other hand, using the stripping phase pH 7, 15% isopropyl alcohol, and 0.8 M KCl, a back extraction efficiency of 83% was obtained. Overall, this study reinforces the potential of reverse micellar extraction as a simple and inexpensive technique for the purification of recombinant proteins [107].

Immunomagnetic microspheres have also been applied for the purification of IFNs, representing a rapid, simple, and target-specific protein separation. To accomplish IFNα-2b purification from crude cell lysates, Yang and coworkers [108] prepared magnetic cellulose microspheres coupled with anti-IFNα-2b monoclonal antibodies. This technique takes advantage of the selectivity of affinity chromatography when coupled with appropriate ligands (such as antibodies), combined with the high availability and efficiency of magnetic response of the microspheres. Size-exclusion HPLC showed that IFNα-2b purified from crude cell lysate had an overall purity of 92.9%, while immunological and biological assays showed an activity recovery of 88.5% and specific antiviral activity of 2.7 × 10^8^ IU/mg. This study illustrated the favorable separation media, combining desired properties for the development of magnetic separation of biological materials [108].

Overall, despite some promising results, the results herein presented demonstrate that alternative techniques for IFNs purification are scarcely studied; therefore, there is still much work to be done to develop efficient alternatives for IFN downstream processing.

### 3.3. Therapeutics and IFN Delivery

Upon administration, many therapeutic proteins exhibit some disadvantages/limitations—namely: (i) low oral and transdermal bioavailability (translates in the need of injections or infusions); (ii) short circulating half-lives, thus requiring a high number of injections; (iii) low aqueous solubility; (iv) high renal clearance rate; (v) capacity to cause local irritation; and (vi) poor stability, resulting from the degradation *in vivo* after administration, which can occur at the administration site or on the protein’s journey to the site of action [111,112,113,114]. Most IFNs, especially those of the α class, are poorly absorbed in the gastrointestinal tract (GIT), also being highly unstable due to the GIT’s acidic pH and high amounts of proteases [112]. Accordingly, their formulations are mainly based on solutions administered parenterally by subcutaneous injection [115]. To improve their pharmacokinetic properties and their clinical utility, several strategies have been designed, including the use of stabilizers/excipients (e.g., carbohydrates, salts, surfactants), chemical coupling of polymers (e.g., PEGylation), and coupling of fusion proteins by genetic engineering (e.g., with human serum albumin-HSA), as well as the encapsulation of therapeutic proteins in drug delivery systems.

#### 3.3.1. Approved Formulations and Excipients

A simple and popular approach to stabilizing and enhancing the solubility of therapeutic proteins is based on the use of multiple excipients, which contribute to reducing aggregation via different mechanisms, such as nonspecific interaction with surface hydrophobic pockets or charged amino acids. Typically, sugars (e.g., mannitol) or salts (e.g., sodium citrate buffer, sodium phosphate buffer) are added to the protein solution. These solutes are thought to be preferentially excluded from the surface of the protein, therefore favoring a compact state [116]. Free amino acids (e.g., arginine, L-methionine, among others) are also often used; the improved performance displayed by arginine at preventing aggregation was additionally demonstrated during the refolding of proteins from inclusion bodies [117]. Polysorbate 20 and 80 (amphipathic, nonionic surfactants) are used in the formulation of biotherapeutic products, both for preventing surface adsorption and as stabilizers against protein aggregation [118]. Along with salts, sugars, and amino acids, protein excipients can also be used as effective stabilizers, as is the case of HSA, which has the following effects: (i) stabilize IFN during shipping and storage; (ii) prevent surface adsorption and aggregation; and (iii) improve solubility, lyophilized cake formation, and dissolving properties of IFN from lyophilized powder [119,120,121]. HSA is extensively used as a stabilizing excipient of proteins because it occurs naturally in the body, is well-tolerated, and has a long half-life—around 19 days [122]. Thus, HSA increases the target protein blood circulation time by protecting the protein from proteolytic degradation while reducing its elimination through the kidneys and ultimately improving the therapeutic efficacy of the biopharmaceutical. Other stabilizers include poloxamers, propylene glycol, and other polymers that sterically hinder protein–protein interactions and limit diffusion. Poloxamers (nonionic polymers) are common excipients used as solubilizing agents and are widely applied in biopharmaceuticals formulations by the pharmaceutical industry [123,124]. An overview of excipients commonly used in IFN formulations is shown in Table 6. The type of formulation—namely, as a liquid or a dry powder—influences the selection of excipients, with sugars, polyols, and amino acids usually being included in powder formulations to ensure the stability of the target biopharmaceutical. In general, the higher propensity to aggregation and surface adsorption by IFNβ-based products (Betaseron^®^ and Avonex^®^) requires a high concentration of HSA than, for instance, IntronA^®^.(Merck Sharp & Dohme Corp, Kenilworth, NJ, USA) Additionally, Peg-IFN products display the longest shelf-lives, and the degradation of Peg-IFN in Pegasys^®^ via oxidation was inhibited by the inclusion of benzyl alcohol [121]. Additional details on IFN formulations, shelf-lives, and special precautions are available in the review by Juppo and co-workers [121].

#### 3.3.2. Chemical Conjugation and Genetically Engineered Fusions

Two approaches extensively used to prolong the half-lives of circulating IFNs and improve drug delivery include chemical conjugation or fusion to specific protein moieties. Chemical modification can be obtained, for example, by PEGylation, in which PEG moieties are attached to the target protein. PEGylation is a well-established procedure in post-production modification methodology to improve the physicochemical properties of IFN. PEGylation of proteins can be performed by chemically reacting a specific chemical functionality within a protein (e.g., the side chains of lysine, histidine, arginine, cysteine, aspartic acid, glutamic acid, threonine, tyrosine, and serine, as well as the N-terminal amino and the C-terminal carboxylic acid groups) with a suitable PEG chain [117,127]. In general, PEGylated formulations improve the pharmacologic characteristics of unmodified IFNs, i.e., (i) increase solubility and stability of IFN by decreasing proteolytic degradation; (ii) reduce renal clearance rate (e.g., by increasing its size above the renal cut-off of 40–50 kDa); (iii) reduce plasma clearance (e.g., by reducing the metabolic degradation and receptor-mediated uptake of the protein from the systemic circulation); (iv) improve the safety profile of the protein by shielding antigenic and immunogenic epitopes; and (v) prolong the circulation time from 5 to 90 h, which is achieved by increasing its molecular size to above that needed for half-life extension [48,128]. A key advantage of using PEGylated proteins is that patients require fewer doses to maintain the necessary therapeutic levels in circulation (less frequent administration), which is achieved by an improvement of its pharmacokinetic profile and results in a decrease of the frequency of side effects [117].

In the early 2000s, four forms of pegylated IFNs became commercially available: Pegasys^®^, PegIntron^®^, ViraferonPeg^®,^ and Plegridy^®^ (Table 1). Pegasys^®^ (Pegferon alpha-2a, Roche) is a therapeutic formulation obtained from the covalent conjugation of recombinant INFα-2b (20 kDa) with a single branched bis-monomethoxy PEG chain (40 kDa). PEG moiety is linked at a single site to the IFNα-2b moiety via a stable amide bond to lysine. This biopharmaceutical is administrated via subcutaneous injection. Furthermore, each vial contains pegylated IFNα-2a, acetic acid, benzyl alcohol, polysorbate, sodium acetate trihydrate, and sodium chloride [125]. PegIntron^®^ (PEGylated IFNα-2b) is available in the form of a white powder. PegIntron^®^ is a covalent conjugate of recombinant IFNα-2b with monomethoxy PEG (12 kDa). Each vial contains pegylated IFNα-2b, disodium phosphate anhydrous, sodium dihydrogen phosphate dihydrate, sucrose, and polysorbate 80, and it is reconstituted in water for injections [125,129]. Plegridy^®^ is a PEG-conjugated form of glycosylated, recombinant IFNβ-1a modified with a single, linear molecule of 20 kDa PEG-O-2-methylpropionaldehyde and is indicated for the treatment of patients with relapsing forms of multiple sclerosis. This biopharmaceutical is administered subcutaneously, and it is sold as a single-use prefilled pen containing pegylated IFNβ-1a, sodium acetate trihydrate, glacial acetic acid, arginine hydrochloride, and polysorbate 20 in water [125]. Notwithstanding the beneficial effect of PEGylation, the low degradability of PEG coupled with potential immunogenicity issues have stimulated research toward alternative serum half-life extenders, such as genetic fusions of IFN molecules to HSA. An example is Albuferon^®^ (Human Genome Sciences, Inc.; Rockville, MD, USA), consisting of a genetic fusion that was developed to enhance the pharmacokinetics of IFN therapy, increasing half-life and maintaining its stabilization. This conjugate results from the fusion between a recombinant IFNα-2b and human albumin. This formulation is well-tolerated and has a prolonged serum half-life that allows dosing at intervals of two to four weeks [125]. In general, both PEGylated and albuminate formulations have consequences in dosage, absorption, bioavailability, and clearance; it is important to know their differences to ensure proper treatment [6].

Currently, some PEGylated IFN formulations are in the pipeline undergoing preclinical and clinical trials [48], including Bolder BioTechnology’s (Boulder, CO, USA) PEGylated IFNβ (completed preclinical testing) and PEGylated IFNα (preclinical development), as well as PharmaEssentia’s (Burlington, NJ, USA) RogPEGinterferonα-2b (P1101) (preclinical development). Although not all the products in progress are glycosylated, glycosylation may enhance the *in vivo* stability of IFN formulations [48].

#### 3.3.3. Drug Delivery Systems

The common route of administration of therapeutic proteins is parenteral injection, which, however, causes pain and has a risk of infection, a high-cost, and low patient compliance. On the other hand, non-invasive drug delivery systems via oral, nasal, pulmonary, ophthalmic, rectal, or transdermal routes may offer significant advantages, including the possibility of self-medication free of needle stick injury, low risk of infection, cost-effectiveness, and better patient compliance [130]. However, the application of “*free*” therapeutic proteins is restricted due to their high instability, namely due to their large molecular size, hydrophilicity, low permeability, rapid elimination from circulation, and high susceptibility to degradation under low pH and in presence of proteases [131]. To overcome this problem, tailored drug delivery systems with controlled particle size and surface modifications have been developed, allowing improvements on the target selectivity, systemic half-life, and bioavailability [130]. A wide range of drug delivery systems have been reported for therapeutic proteins, ranging from polymeric nanocarriers (nanospheres, nanocapsules, micelles), lipid-based nanocarriers (liposomes, solid lipid nanoparticles, nanoemulsions), dendrimers, and hydrogels, among many others.

Given the challenges exposed above, there is thus a need for developing drug delivery systems that protect IFNs from degradation while aiming for extended drug releases. In the early stages, it was believed that if bioavailability was increased by administrating higher doses of the target drug, the treatment efficiency would automatically improve [132]. But such was not the case, since an increment in the commonly applied dose increased toxicity in the central nervous system, while the antitumor efficacy results were not showing the expected incremental improvement. Therefore, the conclusion was that small doses with minimal side effects were more beneficial than higher doses [132].

Over the years, several drug delivery systems, such as liposomes, nanoparticles, microspheres, and gels, among others, have been formulated for the delivery of IFN molecules (Table 7) [133,134].

**Table 7:** Representative studies of IFN drug delivery systems (NR–Not Reported).

**IFN**	**Drug Delivery System**	**Composition**	**Loading (%)**	**Encapsulation Efficiency (%)**	**Release**	**Experimental Conditions**
IFNγ [135]	Microspheres	Poly(lactic-co-glycolic acid) (PLGA)	3.2 (*w/w*)	100	~1.6% ~30–38% in 7 days	
IFNα [136]	Nanoparticles	PLGA/Pegylated PLGA		78–91	90% in 16 days	
IFNα-2b [137]	Microspheres	Poly(ethylene glycal/butylenes terephthalate)-PLGA		86.01	16.7% initial burst 83.1% in 23 days	In vivo
IFNγ [138]	Elastomer	Star-poly(Ɛ-caprolactone-co-D,L-lactide) elastomer	NR	NR	83% in 21 days	BV-2 microglial cells
IFNα [139]	Microspheres	PLGA/poloxamer PLGA/poloxamer blend	NR	NR	2–24% initial burst	Melanoma (A 2058 cells)
IFNα-2b [140]	Hybrid	PLGA Nanoparticles-CS/GP	NR	NR	40% initial burst	In vivo
IFNα-2b [141]	Hydrogel	Hydroxypropyl cellulose	NR	50	50% in 5 h 81% in 24 h 90% in 120 h	Gastric Cancer (MKN-45 cells) Melanoma (A375 cells)
IFNβ [142]	Hydrogels	P(MAA-*g*-EG)	77	NR	40% at pH 1.2 70% at pH 6.8	In vivo
IFNα [143]			60	NR		Colorectal adenocarcinoma (Caco-2 cells) Colon carcinoma (HT29-MTX cells)
IFNα [144]	Bioconjugate	Aldehyde-modified hyaluronic acid				Kidney (VERO cells)
IFNα-2b [145]	Microspheres	Chitosan-carboxymethyl	11	90	7.4% in 1 h 89% in 24 h	Lung adenocarcinoma (A549 cells)
IFNα-2b [146]	Nanoparticles	Chitosan	NR	100	0.5 h 20.5% in pH 1.2 89.6% in pH 6.8	Kidney (MDBK cells)
IFNβ [147]	Nanoparticles	Chitosan/sulfobutylether-β-cyclodextrin	NR	88	87%	
IFNα [148]	Nanoparticles	HSA-IFN-α/poly(sodium-4-styrene) sulphonate/chitosan	76.13	49.1		In vivo
IFNα [149]	Particles	Calcium phosphate	0.2–3.1	80–96	50% in 1 h 80% in 6 h	Cervical cancer (HeLa cells)
IFNα [150]	Liposomes	PEGylated lipids	NR	81	30% in 8 h	Vaginal tissue
IFNβ [151]	Microparticles	Trimethyl-chitosan (TMC), poly(ethylene glycol)dimethacrylate (PEGDMA) and methacrylic acid (MAA)	53.25			In vivo
IFNα-2b [152]	multivesicular liposome	DOPC, cholesterol, DPPG, triolein	30			In vivo
**Clinical Trials**						
**IFN**	**Drug Delivery System**	**Composition**		**Indication**		
IFNα-2b [153]	HeberPAG^®^	Sodium phosphates, Dextran-40, kalium phosphate, sodium chloride, kalium chloride, mannitol, saccharose, and human albumin		Mycosis fungoides		
IFNγ [154]	CIGB-128-A	Trehalose, succinic acid and human serum albumin		Potential application in several malignancies		
INF-α2b [155]	Microspheres	Gelatin, a cationic arginine-rich protein stabilizer, protamine sulphate		Ovarian cancer (SKOV3 cells)		
INF-α2b [156]	Locteron	Poly(ether-ester) microspheres		Hepatitis C therapy		

Indeed, the development of new IFN delivery strategies is a key issue for simplifying its administration and improving its therapeutic effects, but also reducing its dose-related side effects without decreasing their biological activity or changing the structure of the biotherapeutic material [120,133]. One of the most attractive approaches toward this aim is the encapsulation of IFN into poly(lactic-*co*-glycolic acid) (PLGA) microspheres. In 1997, Yang and Cleland [135] developed a formulation by microencapsulation of IFNγ in PLGA microspheres using the *water-oil-water* technique. The loading of IFN*γ* in the microspheres was 3.2% (*w/w*) and the encapsulation efficiency was 100%. In vitro release studies showed an initial burst of ~1.6%, with a cumulative release of 30–38% at day 7. The continuous release of protein from microspheres should occur based on the diffusion of protein out of the eroding microspheres over time. However, potential electrostatic interactions between IFNγ (isoelectric point > 9.5) and the acidic end groups generated by the hydrolysis of PLGA may prevent the release of positively charged IFNγ at physiological pH. These studies indicate that IFNγ did not adsorb to PLGA, but there was adsorption of IFNγ (~25 µg) to the nitrocellulose filter device used in release studies. The effect of the components in the buffer system on the release of rhIFNγ from the microspheres was then investigated. Different release profiles were observed with different buffers. A high salt concentration (100 mM), high osmotic strength (40 mg/mL mannitol), or low SDS concentration (0.01% *w/w*) was not suitable for the in vitro release studies because aggregation and/or precipitation of IFNγ occurred in each case. The pH and type of buffering species also affected the release of IFNγ. The buffer pH and buffer species had a direct impact on the differential solubility, stability, and aggregation of IFNγ. This study identified that the stability of IFN released from these microspheres is one of the most important concerns about the therapeutic potential of this approach [135]. Being conscious of this problem, Alonso and coauthors [139] developed new delivery strategies for the encapsulation of IFNα into biodegradable micro- and nanospheres. IFNα was encapsulated within PLGA/poloxamer 188 blend microspheres prepared by an *oil-in-oil* solvent extraction technique or within PLGA micro- and nano-spheres containing poloxamer, prepared by the *water-in-oil-in-water* solvent evaporation technique. Poloxamer 188 was used as a stabilizing agent. The findings demonstrated that both techniques led to an efficient encapsulation of IFNα and modulation of its particle size, which ranged from 280 nm—PLGA nanospheres containing poloxamer—to 40 µm—PLGA/poloxamer blend microspheres. Additionally, these systems exhibited a release pattern that was characterized by an initial burst (2–24% IFNα) followed by small pulses of immunoenzymatically detected IFNα for up to 1 month. In vitro studies showed that the antiproliferative activity of the IFNα varied depending on the formulation. Specifically, PLGA/poloxamer blend microspheres were able to provide significant amounts of active IFNα for up to 96 days. More recently [140], biodegradable PLGA nanoparticles (NPs) containing IFNα-2b were loaded on a chitosan/glycerophosphate (CS/GP)-based thermosensitive hydrogel for IFN delivery by intratympanic injection. The injectable hydrogel exhibited a rapid transition from solution to semi-solid gel as temperature increased (37 °C), presented a porous structure and displayed a long-term release profile in vitro. Owing to the properties of PLGA NPs and in situ hydrogel, PLGA NPs-CS/GP tended to reduce drug clearance and extended the residence time in the inner ear, after which a continuous and consistent release of the drug in the cochlea was observed. In the guinea pig cochlea, a 1.5- to 3-fold increase in the drug exposure time of PLGA NPs-CS/GP was observed, in comparison with those of the solution, PLGA NPs, and IFN-loaded hydrogel. Most importantly, a prolonged residence time was attained without obvious histological changes in the inner ear. This biodegradable, injectable, and thermosensitive PLGA NPs-CS/GP system may allow longer delivery of protein drugs to the inner ear, and thus represents a potential novel vehicle for inner ear therapy. In general, PLGA-based delivery systems opened new avenues for improving IFN-based therapies.

Amsden and coauthors [138] demonstrated the sustained delivery of IFNγ from a photocrosslinked biodegradable elastomer. The elastomer was prepared through the UV initiated crosslinking of end terminal acrylated *star*-poly(ε-caprolactone-*co*-_D,L_-lactide). Bioactive IFNγ was released from the optimum formulation at a constant rate of 23 ng/day over 21 days (total release of IFNγ over 83%). These results represent an improvement over previously published results for IFNγ release from any type of formulation [138]. As noted above, Yang and Cleland [135] only achieved a retention of biological activity of IFNγ in the range 30–38% after 1 week from a PLGA microsphere formulation. Thus, this elastomer-based formulation may be clinically useful for sustained and local protein drug delivery applications. In 2020, Liu and collaborators [141] prepared cross-linkable hydroxypropyl cellulose hydrogels by irradiation techniques. About 50% of the encapsulated IFNα-2b was released within the first 5 h. The burst pattern might have originated from IFNα-2b abrupt release of the exposed drug on the surface of the hydrogels. Subsequently, IFNα-2b release from the hydrogels continued at a much slower release rate for a long period through diffusion mechanism from the porous network. It was found that 80.91 ± 3.75% of IFNα-2b was released from hydrogels in the first 24 h. In vitro studies demonstrated that IFNα-2b-loaded hydrogels could sensitize T cells against gastric cancer cells, involving the upregulation of the early activation marker CD69 and the secretion inflammatory cytokine-IFNγ. Additionally, the antitumor activity of IFNα-2b-loaded hydrogels combined with CIK (cytokine-induced killer) cells and radiation was evaluated in an MKN-45 xenografted nude mice model. Such *in vivo* assays showed that hydrogels kept the activity of IFNα-2b and allowed the stable release of IFNα-2b to stimulate T cells for a longer time, compared with free IFNα-2b injection or T cells alone. At the same time, low-dose irradiation promoted T cell accumulation and infiltration in subcutaneous tumors. This innovative integration mode of IFNα-2b-loaded hydrogels and radiotherapy offers a potent strategy to improve the anticancer effects of T cells on gastric cancer [141].

Takayama and coauthors [142] reported the preparation of poly(methacrylic acid-*g*-ethylene glycol) P(MAA-*g*-EG) hydrogels and their subsequent application as suitable carriers to improve the intestinal absorption of IFNβ. P(MAA-*g*-EG) hydrogels exhibited high loading efficiency for IFNβ (77%). Moreover, IFNβ-loaded P(MAA-*g*-EG) hydrogels showed a pH-sensitive release behavior. In the first hour, the release of IFNβ from hydrogels at pH 1.2 condition was approximately 40%, which was lower than the release efficiency at pH 6.8 (~70%). Furthermore, a drastic reduction of plasma calcium concentration accompanied by calcium absorption and a dose-dependent enhancement of plasma IFNβ concentration were observed after the administration of particles loaded with IFNβ into closed rat ileal segments. Overall, the administration of IFNβ using these hydrogels significantly improved the intestinal absorption of IFNβ. pH-sensitive hydrogels were also used for delivery of IFNα [143]. The biophysical mechanisms controlling the transport of IFNα were investigated using a Caco-2/HT29-MTX co-culture as a GIT model. The synthesized nanoparticles exhibited pH-responsive swelling behavior and allowed the permeation of IFNα through the tight junctions of the developed cellular gastrointestinal epithelium model. Both studies demonstrate that P(MAA-*g*-EG) hydrogels are promising carriers for oral delivery of IFN.

In 2016, the Rosato research group [144] reported a bioconjugate composed of hyaluronic acid (HA) and IFNα-2a. The conjugation with HA did not substantially modify either the antiviral function or the anti-proliferative activity of the cytokine. Moreover, the induction of STAT1 phosphorylation and of a specific gene expression signature in different targets was retained. In vivo studies in ovarian cancer xenograft mouse models showed that HA-IFNα-2a bioconjugate exhibited a superior antitumor activity without being toxic for intraperitoneal organs in comparison with the free IFNα-2a. Overall, HA-IFNα-2a bioconjugate disclosed an improved anticancer efficacy and can be envisaged as a promising loco-regional treatment for ovarian cancer [144].

Over the years, pharmaceutical development of drug delivery system has been pursued enthusiastically by many scientists. To date, promising results have been accomplished with respect to improved drug delivery systems, envisaging administration of IFNs in a less invasive and safer manner with reduced frequency, reduced immunogenicity, and therefore better patient compliance. Additional investigation of IFN pharmacokinetics and treatment efficacies of these novel systems may pave the way for their acceptance by the pharmaceutical industry.

## 4. Outlook and Future Prospects

The approval of protein-based biopharmaceuticals in the beginning of the 1980s has led to major improvements in overall health and quality of life. IFNs are crucial elements of cellular defense mechanisms in humans and have demonstrated their clinical effectiveness against viral infections, cancer, and neurodegenerative diseases, respectively, by limiting virus replication, reducing tumor cell mass, or by controlling disease symptoms and prolonging survival. Type I α-IFN was the first biotherapeutic approved (1986), which then paved the way for the development of IFNβ- and IFNγ-based products. In addition to the administration of IFNs as single agents, their introduction in combined treatment regimens (e.g., with ribavirin) have also demonstrated promising clinical outcomes. Since 1986, 22 distinct IFN formulations have been approved by regulatory agencies, of which three have been withdrawn from the market. The current 172 active clinical trials involving IFNs reinforce their importance as human health biotherapeutics. The overall market sales of IFNs reached US$6.9 billion in 2019, and these numbers are projected to grow in the near future owing to the increasing incidence of chronic diseases and the increasing adoption of biosimilars for possible therapeutics or prophylaxis of future pandemics, among others.

Stimulated cells were initially the main sources of IFNs; however, the remarkable developments in the technology of recombinant DNA have rapidly led bacterial-derived IFNs to dominate the market. Currently, most commercial IFN products are obtained from *E. coli*, with the exception of two IFNα formulations that are obtained from leukocytes and lymploblastoid cells, as well as IFNβ-1a, which is obtained from CHO cell lines. Four main reasons may explain why *E. coli* took the edge for the cost-effective and high-yield production of IFNs: (i) the first one is associated with IFNs, namely their low molecular weight and, at least in some cases, the lack of extensive post-translational modifications such as glycosylation that are needed to obtain bioactive molecules; (ii) *E. coli* was one of the first hosts to be used in recombinant DNA technology; (iii) a wide range of *E. coli* molecular tools (improved strains, vectors, and promoters) became highly accessible; and (iv) the inherent *E. coli* advantages (fast growth kinetics in simple and inexpensive media) coupled with the intensive investigation of *E. coli* recombinant systems allowed the identification of optimized culture conditions (culture media, cultivation strategies including improved induction regimens) and genetic strategies (e.g., removal of codon bias), thus favoring high expression of IFNs. Notwithstanding the high success displayed by *E. coli*, it was here overviewed that IFN secretion to a culture medium using *P. pastoris* can deliver higher quantities of bioactive IFNs with a lower level of contaminants. Although in the case of IFNs, this has not translated to industry since no commercial IFN biotherapeutics derived from *P. pastoris* are currently marketed; nonetheless, this system has been successfully used to obtain protein-based products with clinical utility. Commercial IFNβ-1a is obtained using CHO cell lines, and the rationale behind this choice is that unlike most IFNs, glycosylated IFNβ-1a may display an improved bioactivity *in vivo*, which is not achievable using *E. coli*.

With regard to the first step of the downstream processing of IFNs, specific strategies have been applied for the extraction and isolation of IFNs, which depend on the host. Using *E. coli*, mild cell disruption methods, using for instance osmotic shock, are crucial for obtaining selective product release from periplasm while avoiding contamination from soluble host cell proteins. *E. coli* disruption can be efficiently achieved using high-pressure homogenization or sonication, and eukaryotic proteins tend to accumulate as inclusion bodies without the use of solubility enhancers, which require additional processing steps. It has been demonstrated that washing inclusion bodies using low concentrations of urea, the non-ionic detergent triton X-100, or sodium deoxycholate can improve purity before the final solubilization step with urea or guanidine hydrochloride, often enhanced at alkaline pH and in which a reducing agent can be added to prevent the formation of non-native disulfide bonds. As proteins are generally recovered in a biologically inactive form from inclusion bodies, the final step includes their refolding, which can be carried out by the addition of denatured protein solution to refolding buffer or by chromatography (e.g., by SEC, IEX, HIC, IMAC) whereby simultaneous purification occurs. Supplementation of refolding buffer with sugars, reducing agents, amino acids, or reduced and oxidized glutathiones can improve the refolding yield. As with *E. coli*, intracellular *P. pastoris* heterologous proteins can be recovered after the application of high-pressure homogenization, while secreted proteins are generally subjected to dialysis, concentration steps, or microfiltration to remove medium culture components. During this process, inclusion of detergents, sugars, or NaCl contributes to preventing aggregation of IFNs. Distinct capture and polishing chromatographic steps have been explored for IFNs purification. Although reverse-phase chromatography may negatively impact the bioactivity recoveries of IFNs, purification of fusion proteins by IMAC is extensively used with success; however, since the protein is produced using non-native amino acids and its removal requires additional processing steps, IMAC’s use is generally restricted to obtain IFNs for structural studies. IEX, HIC, and SEC generally allow efficient purification of recombinant IFNs, and as expected, multi-step chromatography allows IFN purities higher than 98% to be obtained. Alternative purification methodologies to chromatography have also been described, which, however, are generally less efficient than chromatography. In this regard, ATPS have gathered much attention, although rather than an intermediate purification step, its application in capture and clarification of culture broths/cell lysis supernatants seems particularly promising.

As with other therapeutic proteins, IFNs may have short half-lives and are rapidly degraded, thus requiring multiple administrations. A common problem associated with IFN therapeutics is that they are required over long periods, inducing immune responses in the host, and ultimately decreasing their therapeutic efficacy. Aiming to improve the pharmacokinetic properties of IFN biopharmaceuticals while reducing potential immunogenicity problems, several strategies have been designed, including the addition of excipients to IFN formulations, coupling of polymers or proteins, as well as IFN encapsulation in drug delivery systems. IFNs administration is mostly performed via subcutaneous, intramuscular, intravenous, or intralesional injection, and are formulated with polysorbates, salts, HSA, sugars, and poloxamers, among others. PEGylation and albumination, respectively achieved by chemical coupling of PEG moieties or genetically fusing HSA to IFNs, have also been explored in clinical formulations, allowing a decrease in the number of injections and a reduction of the side effects. In addition, some drug delivery systems have been engineered, envisaging controlled release of bioactive IFNs by using different formats—hydrogels, nanoparticles, microspheres, or liposomes—and making use of different polymers—namely, PLGA and chitosan. The expected emergence of biosimilars in the coming years due to the expiration of patents for the original biopharmaceuticals will create an opportunity for taking advantage of the expertise and knowledge acquired in the last decades and the breakthroughs herein discussed.

Overall, the manufacturing of recombinant IFNs was overviewed, demonstrating the major advances achieved in the field. So far, more than twenty IFN-based formulations have received regulatory approval, accounting for significant market share and which, due to their multiple therapeutic actions still being investigated in clinical trials, are projected to have a role in improving human health.

## Figures and Tables

**Table 1:** Therapeutic interferons approved in the United States of America (USA) and European Union (EU).

Interferon (IFN) Type/Subtype		Clinical Indication	Commercial Name	Active Pharmaceutical Ingredient	Approval Date
IFNα (I)	IFNα-2a	Hairy cell leukemia; AIDS-related Kaposi’s sarcoma; Chronic myelogenous leukemia; Cutaneous T-cell lymphoma; Chronic hepatitis B and C; Follicular lymphoma; Malignant melanoma	Roferon A^®^ Hoffmann–La Roche (Basel, Switzerland)	IFNα-2a (*E. coli*)	1986 (EU) 1986 (USA)
		Chronic hepatitis B; Chronic myelogenous leukemia; Melanoma	Pegasys^®^ Hoffmann–La Roche (Basel, Switzerland)	PEGylated IFNα-2a (*E. coli*)	2002 (USA and EU)
	IFNα-2b	Multiple myeloma; Chronic myelogenous leukemia; Chronic hepatitis B and C; Carcinoid tumor; Hairy cell leukemia; Follicular lymphoma; Malignant melanoma; Condylomata acuminate; Kaposi’s sarcoma	Intron A^®^, Alfatronol^®^ (Merck Sharp & Dohme Corp., Kenilworth, NJ, USA)	IFNα-2b (*E. coli*)	1986 (USA) 1986 (EU)
		Chronic hepatitis B and C	Viraferon^®^ (Schering-Plough Corporation, Brussels, Belgium)	IFNα-2b (*E. coli*)	2000 (EU)
		Chronic hepatitis C	Rebetron^®^ (Schering-Plough Corporation, Brussels, Belgium)	ribavirin/IFNα-2b (*E. coli*)	1999 (USA)
		Chronic hepatitis C	ViraferonPeg^®^ (Merck Sharp & Dohme Corp., Kenilworth, NJ, USA)	PEGylated IFNα-2b (*E. coli*)	2000 (EU)
		Chronic hepatitis C	PegIntron^®^ (Schering-Plough Corporation, Brussels, Belgium)	PEGylated IFNα-2b (*E. coli*)	2001 (USA) 2000 (EU)
		Chronic hepatitis C	Albinterferon^®^/Albuferon^®^ (Novartis—Basel, Switzerland; Human Genome Sciences, Rockville, MD, USA)	Fusion protein of albumin and IFNα-2b (*E. coli*)	2010 (USA)
		Melanoma	Sylatron™ (Merck & Co., Inc, Kenilworth, NJ, USA)	PEGylated IFNα-2b (*E. coli*)	2011 (USA)
	IFNα-2c	Chronic viral hepatitis; HIV infection	Berofor^®^ (Boehringer Ingelheim, Lda, Ingelheim am Rhein, Germany)	IFNα-2c (*E. coli*)	1989 (USA)
IFNα (I)	IFNα-n3	Condyloma acuminate	Alferon N^®^ AIM ImmunoTech (Philadelphia, PA, USA)	IFNα-n3 *(human leukocytes)*	1987 (USA)
	IFNα-n1 (lymphoblastoid)	Chronic hepatitis B and C; Hairy cell leukemia; HPV infection	Wellferon^®^Glaxo Wellcome (London, United Kingdom)	IFNα-n1 *(human lymphoblastoid cells)*	1997 (USA)
	IFNα-con-1	Chronic hepatitis C	Infergen^®^ (Three Rivers Pharmaceuticals, Warrendale, USA)	IFNα (*E. coli*) IFNα + Ribavirin (*E. coli*)	2001(USA)
IFNβ (I)	INFβ-1a	Multiple sclerosis	Avonex^®^ (Biogen Idec, Maidenhead, United Kingdom)	IFNβ-1a *(CHO cells)*	1996 (USA) 1997 (EU)
			Rebif^®^ (EMD Serono, London, United Kingdom)	Glycosylated IFNβ-1a *(CHO* cells*)*	2002 (USA) 1998 (EU)
			Plegridy^®^ (Biogen Idec, Maidenhead, United Kingdom)	PEGylated IFNβ-1a *(CHO)*	2014 (EU and US)
	INFβ-1b	Multiple sclerosis	Betaseron^®^ (Chiron—Emeryville, USA; Berlex Laboratories, Richmond, VA, USA)	IFNβ-1b (differs from human protein in that Cysteine-17 is replaced by Serine) (*E. coli*)	1993 (USA)
			Betaferon^®^ (Bayer Pharma, Leverkusen, Germany)		1995 (EU)
			Extavia^®^ (Novartis Europharm, Camberley, United Kingdom; Novartis Pharmaceuticals, East Hanover, NJ, USA)	IFNβ-1b (*E. coli*)	2008 (US) 2009 (EU)
IFNγ (II)	INFγ-1b	Chronic granulomatous disease; Osteopetrosis	Actimmune^®^ (Vidara Therapeutics, Dublin, Ireland)	IFNγ-1b (*E. coli*)	1990 (US)
			Imukin^®^ (Boehringer Ingelheim, Lda, Ingelheim am Rhein, Germany)		1996 (US)

Abbreviations: CHO–Chinese hamster ovary; *E. coli*–*Escherichia coli*. Note: Data taken from [3,13,14].

**Table 2:** Classification of interferons based on the type of receptor through which signaling takes place. Adapted from Diamond and collaborators [21].

IFN Type	Class	Discovery Year	Receptor Binding
I	α	1957	High binding affinity to IFNAR2, which then recruits low-affinity IFNAR1 to form the signaling competent ternary complex
	β	1957	
	ω	1985	
	τ	1996	
II	γ	Early 1970s	Affinity for IFNGR (IFNGR1 and IFNGR2)
III	λ1	2003	High binding affinity to IFNLR1, which then recruits low-affinity IL-10Rβ to form signaling competent ternary complex
	λ2		
	λ3		
	λ4		

**Table 6:** Overview of formulation excipients commonly used in IFN products [121,125,126].

Excipient		Proposed Role	IFN Formulations	Highlights
Buffers	Sodium phosphate pH 7	Adjust pH to maximize the conformational stability of IFNs	IntronA^®^, PegIntron^®^, ViraferonPeg^®^, Alferon N^®^	Contrary to IFNα-2a, biological activity of IFNα-2b is high at pH 7; Acetate is not suitable for dry products due to the volatility of acetate and changes in pH during lyophilization
	Acetate buffer pH 5		Roferon^®^	
	Acetate buffer pH 6		Pegasys^®^	
Surfactants	Polysorbate 20	Inhibit protein aggregation and adsorption to surfaces	ViraferonPeg^®^, Imukin^®^	Widely used independent of the type of IFN
	Polysorbate 80		Roferon^®^, ViraferonPeg^®^, Pegasys^®^	
	Poloxamer 188		Rebif^®^	
Chelating agents	Edetate disodium	Mitigate risk of oxidation and immunogenicity from aggregates	IntronA^®^	
Salts	NaCl	Tonicity modifier	IntronA^®^, Roferon^®^, Pegasys^®^	Liquid formulations
Sugars and polyols	Sucrose	Lyoprotectant and tonicity modifier	ViraferonPeg^®^	Powder formulation
	Mannitol	Lyoprotectant	Actimmune^®^, Immukin^®^	
Preservatives	Benzyl alcohol	Oxidation inhibition	Pegasys^®^	
Proteins	Human serum albumin	Prevents aggregation	IntronA^®^, Betaseron^®^	Higher albumin concentrations if IFNβ products due to their higher tendency to aggregate than IFNα-based products
Amino acids	Arginine	Increase protein solubility and stability and preserves biological activity	Avonex^®^	Often used as an alternative to albumin
	Glycine	Prevents aggregation	Betaseron^®^	
